# Influence of Nano-TiO_2_ Particle Size on Fresh, Physical, Mechanical, Photocatalytic, and Cost Performance of Cement Mortars

**DOI:** 10.1021/acsomega.6c02257

**Published:** 2026-04-15

**Authors:** Zehra Funda Akbulut

**Affiliations:** Department of Mining Engineering, Faculty of Engineering, 53000 University of Van Yüzüncü Yıl, Van 65080, Türkiye

## Abstract

Nanosized additives have attracted significant attention for improving cement-based materials; however, the isolated effects of nano-TiO_2_ (NT) particle size and dosage on engineering performance and photocatalytic efficiency remain unclear. This study investigates NT with particle sizes of 15–35 nm and 55–75 nm incorporated at 1–3% replacement levels in cement mortars at a constant water-to-binder ratio of 0.5. NT reduced the flow diameter from 225 mm (control) to 209 mm at 3% (15–35 nm), confirming greater workability loss for finer particles. Water absorption and apparent porosity decreased from 8.62% and 10.12% to 8.12% and 9.53%, respectively. Compressive strength increased from 44.57 to 47.21 MPa at 2% NT (15–35 nm), while flexural strength remained stable (4.13–4.27 MPa). Photocatalytic activity improved markedly: ΔE values increased from 3.54 (control, 4 h) to 6.76 for 2% NT (15–35 nm), representing a 91% enhancement. Larger particles showed lower efficiency. The results indicate that 2% NT provides the optimal balance between mechanical improvement, photocatalytic performance, and cost efficiency.

## Graphical abstract

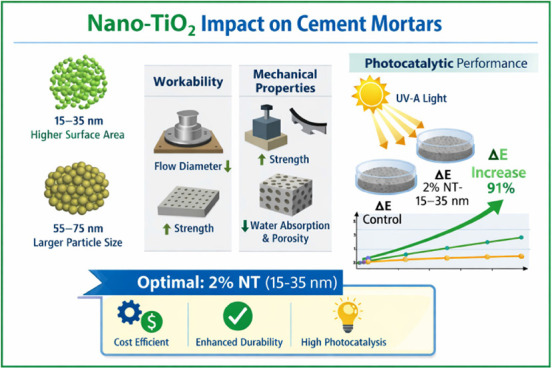

## 1 Introduction

Cement-based materials remain the most widely used construction materials worldwide due to their versatility, availability, and cost-effectiveness.^1^ However, the production of Portland cement (PC) is highly energy-intensive and is responsible for a considerable share of global anthropogenic CO_2_ emissions.^2^ These environmental concerns have accelerated efforts to develop more sustainable cementitious systems that reduce cement consumption while preserving or enhancing engineering performance.^3^ In this regard, advanced material modification strategies that improve performance efficiency without increasing binder demand have attracted significant research interest.^4^

Among emerging approaches, the incorporation of nanoscale materials into cementitious matrices has proven to be a promising route for performance enhancement aligned with sustainability goals.^5^ Owing to their exceptionally high specific surface area (SSA) and size-dependent physicochemical characteristics, nanomaterials substantially influence hydration mechanisms.^6^ They provide additional nucleation sites for hydration products,^7^ promote pore refinement through microfiller effects,^8^ and enhance matrix homogeneity.^9^ Consequently, improvements in fresh-state behavior,^10^ reductions in transport-related parameters such as water absorption (WA) and apparent porosity (AP),^11^ and enhancements in mechanical properties^12^ have been widely reported. Nanomodification is therefore increasingly recognized as a pathway toward next-generation high-performance cement-based materials.

Among various nanomaterials, nanotitanium dioxide (NT) has attracted particular attention due to its chemical stability, compatibility with cement systems, and pronounced influence on microstructural development.^13^ The high SSA of NT facilitates strong interactions with hydration products, accelerating hydration kinetics and contributing to a denser matrix structure.^14^ While NT is extensively studied for its photocatalytic functionality,^15^ its role as a microfiller^16^ and hydration regulator^17^ has become equally relevant in both structural and functional cementitious applications. Importantly, employing NT as a partial replacement for PC, rather than as an additive, represents a more sustainable strategy by directly reducing cement consumption and associated CO_2_ emissions,^18^ while enabling clearer evaluation of NT’s intrinsic effects under constant binder conditions.

Despite the growing body of research on NT-modified cement mortars, several limitations remain. Many existing studies adopt additive-based approaches that increase total binder content,^19^ potentially obscuring sustainability benefits^20^ and complicating interpretation of performance gains.^21^ Furthermore, prior research frequently focuses on isolated performance indicators, such as compressive strength or durability, without simultaneously evaluating fresh-state properties, pore-related physical parameters, and mechanical behavior at different curing ages. Comprehensive investigations examining the concurrent development of compressive strength (CS) and flexural strength (FS) at both early and later ages, as well as a direct comparison of NT particle sizes under identical mixture proportions, remain limited.

## 2 Research Significance and Novelty

Numerous studies have explored the incorporation of NT into cement mortars, with emphasis on strength enhancement, durability improvement, and selected functional properties. However, most existing investigations rely on a single NT particle size, relatively high replacement levels, or hybrid nanoadditive systems, which limits the ability to interpret particle-size- and dosage-dependent effects. Furthermore, microstructural assessments are often qualitative, and the economic implications of NT incorporation are rarely considered.

The novelty of the present study lies in its systematic, controlled experimental framework, in which NT with two distinct particle-size ranges (15–35 nm and 55–75 nm) is incorporated individually (without hybridization) at replacement levels of 1–3% by weight of cement. All mixtures were produced using a replacement-based approach, with a constant binder content, a constant water-to-binder ratio, and a regulated superplasticizer dosage, thereby enabling an unbiased evaluation of the effects of NT size and superplasticizer dosage.

This study integrates fresh-state workability, pore-related physical properties, mechanical performance, photocatalytic self-cleaning behavior, and economic considerations within a unified framework. Compressive and flexural strengths were evaluated at 3, 7, and 28 days to capture both early-age and long-term responses. Microstructural mechanisms governing macroscopic behavior were examined using scanning electron microscopy (SEM), with quantitative image analysis performed in ImageJ. In addition, photocatalytic self-cleaning performance was assessed via methylene blue discoloration under UV-A irradiation (365 nm), with surface color variations quantified using CIE Lab* measurements. A simplified cost estimation further enhances the practical relevance by correlating performance improvements with material costs. Overall, this work provides a comprehensive, performance-oriented contribution to understanding NT-modified cement mortars from both technical and application perspectives.

## 3 Materials and Methodology

### 3.1 Materials

Ordinary Portland cement (CEM I 42.5 R) conforming to EN 197-1 was used as the primary binder in all mortar mixtures. The cement has a specific gravity of 3.14 g/cm^3^ and a Blaine fineness of 3572 cm^2^/g, which fall within the typical range for this strength class. The use of CEM I 42.5 R ensured consistent hydration behavior and reliable early-age strength development, providing a stable reference matrix for evaluating the influence of nanotitanium dioxide incorporation. The physical, chemical, and mechanical properties of the cement are presented in Table 1.

**Table 1:** Physical, Chemical, and Mechanical Characteristics of Portland Cement (PC)

Chemical properties	
SiO_2_	21.58%
Al_2_O_3_	5.96%
Fe_2_O_3_	2.84%
CaO	60.92%
MgO	2.93%
SO_3_	2.96%
Na_2_O	-
K_2_O	0.78%
CI	0.012%
Physical properties	
Initial setting time	153 min
Final setting time	223 min
Compressive strength (MPa)	
After 2 days	20.28
After 7 days	42.11
After 28 days	51.78

NT was utilized as the nanoscale modifier. Two distinct nano-TiO_2_ (NT) powders with nominal particle size ranges of 15–35 nm and 55–75 nm were used to investigate particle-size-dependent effects. The physical and chemical properties of the NT materials, as provided by the manufacturer (Nanografi Co.), are summarized in Table 2.

**Table 2:** Physical and Chemical Properties of NT Powders as Provided by the Manufacturer

Property	NT-1	NT-2
Nominal particle size	15–35 nm	55–75 nm
Appearance	White powder	White powder
Crystal structure	Anatase phase	Anatase phase
Purity	≥99%	≥99%
Specific surface area (BET)	120–180 m^2^/g	40–70 m^2^/g
Density	∼3.9 g/cm^3^	∼3.9 g/cm^3^
pH (4% aqueous dispersion)	6–8	6–8
Moisture content	<0.5%	<0.5%
Morphology	Nearly spherical nanoparticles	Nearly spherical nanoparticles

The selected particle sizes are intended to create a distinct contrast between SSA and dispersion behavior, while adhering to the range commonly reported for cementitious applications. Figure 1a and b displays the photographic representation of the two different sizes of NT materials.

**Figure 1:**
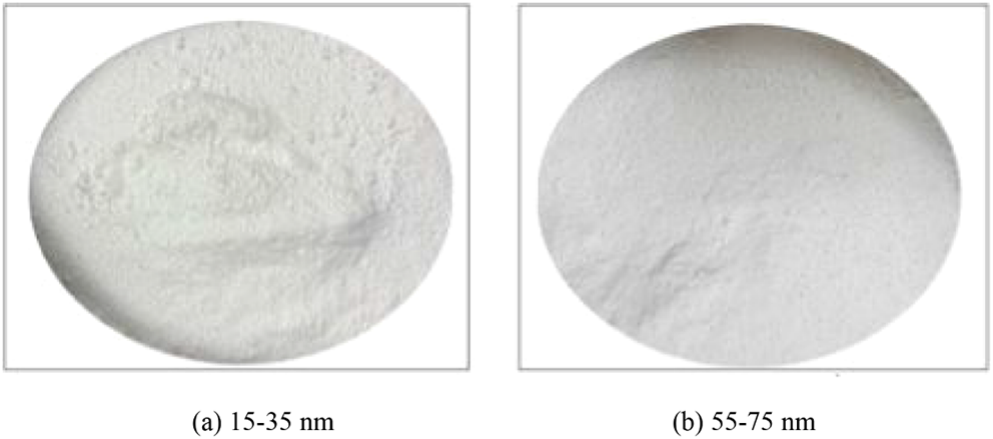
Photographs of the 15–35 nm and 55–75 nm NT materials.

Standardized silica sand with a particle size range of 0–2 mm was used as the fine aggregate in all mortar mixtures. The sand conforms to EN 196-1 specifications and is characterized by a well-defined particle size distribution, a specific gravity of approximately 2.60–2.65 g/cm^3^, and negligible water absorption (≤0.5%). Because the sand is standardized, its physical properties are predefined and consistent; therefore, additional characterization tests were not required within the scope of this study. Standard siliceous sand with controlled grading was used as the fine aggregate in all mixes to ensure consistency and reduce variability in aggregate properties. Tap water was utilized for mixing and curing. A polycarboxylate-based superplasticizer (SP) was used to enhance workability and facilitate the proper dispersion of NT particles within the cement matrix. The optimal SP dosage for each mixture was determined using a trial-and-error method, accounting for NT particle size and replacement level, and was selected to yield manageable mixtures with minimal segregation or bleeding. The particle size distribution of the standardized silica sand (0–2 mm) used as the fine aggregate was defined in accordance with EN 196-1 specifications. Methylene blue (MB) was used as the organic dye for photocatalytic evaluation. The dye was obtained from Sigma-Aldrich (Merck, Germany). The cumulative sieve analysis curve of the standard sand is presented in Figure 2. As shown in Figure 2, the sand exhibits a well-graded particle-size distribution within the specified limits, ensuring consistent packing density and repeatable fresh and hardened-mortar behavior.

**Figure 2:**
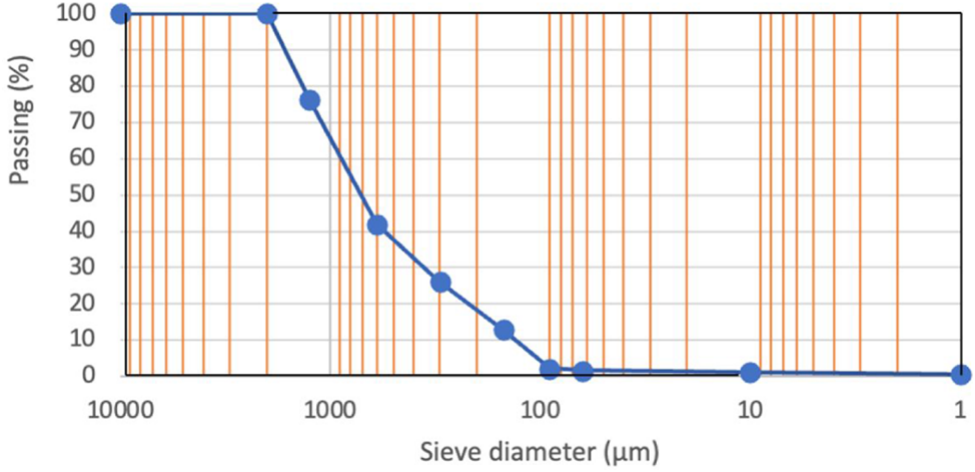
Particle size distribution of standardized silica sand used in the study according to EN 196-1.

### 3.2 Mixture Proportions and Experimental Design

All mortar mixtures were formulated with a constant total binder content and a fixed water-to-binder ratio (w/b) of 0.50 to isolate the effects of NT particle size and dosage. NT was incorporated as a partial replacement of Portland cement (PC) at three dosage levels (1%, 2%, and 3% by weight of total binder). A control mixture without NT (P0) was also prepared. Table 3 presents the detailed mixture proportions and the experimental matrix.

**Table 3:** Mixture Proportions of P0–P6 Mixtures

Mix ID	Nano-TiO_2_ size	Nano-TiO_2_ (%)	Cement (g)	Nano-TiO_2_ (g)	Sand (g)	Water (g)	w/b	SP (% binder)	SP (g)
P0	–	0	450.0	0.0	1350	225	0.50	0.10	0.45
P1	15–35 nm	1	445.5	4.5	1350	225	0.50	0.12	0.54
P2	15–35 nm	2	441.0	9.0	1350	225	0.50	0.16	0.72
P3	15–35 nm	3	436.5	13.5	1350	225	0.50	0.20	0.90
P4	55–75 nm	1	445.5	4.5	1350	225	0.50	0.12	0.54
P5	55–75 nm	2	441.0	9.0	1350	225	0.50	0.16	0.72
P6	55–75 nm	3	436.5	13.5	1350	225	0.50	0.20	0.90

### 3.3 Mixing and Specimen Preparation

The mixing procedure was kept identical for all mixtures to ensure consistency and reproducibility.^22^ Initially, Portland cement (PC) and standard sand were dry-mixed for 2 min to obtain a homogeneous blend. Subsequently, half of the mixing water containing the predetermined superplasticizer (SP) dosage was gradually introduced under continuous mixing. The remaining water was then added to complete the wet-mixing stage.

For the NT-modified mixtures, nanotitanium dioxide (NT) was predispersed in the mixing water prior to mortar preparation to minimize particle agglomeration and promote uniform nanoscale distribution. Dispersion was performed using a probe-type ultrasonic sonicator (QSONICA Q55, 1/8″ probe) operating at 20 kHz with a nominal power of 750 W. The sonicator was set to 60% amplitude and operated in pulse mode (5 s on, 5 s off) for a total duration of 10 min. To prevent overheating and possible alteration of nanoparticle surface characteristics, the suspension temperature was maintained at 20–25 °C using an ice bath throughout sonication. The NT dosage, calculated as a percentage of total binder mass, was fully dispersed in the mixing water and immediately incorporated into the mortar mixture. After NT addition, mixing was continued for an additional 3 min to ensure homogeneous distribution.

Prismatic specimens with dimensions of 40 mm × 40 mm × 160 mm were cast for all experimental evaluations. After casting, the specimens were compacted to eliminate entrapped air, sealed to prevent moisture loss, and stored under laboratory conditions for 24 h. Following demolding, the samples were cured in water at a controlled temperature until the designated testing ages. The preparation, casting, curing, and demolding of the mortar specimens are shown in Figure 3.

**Figure 3:**
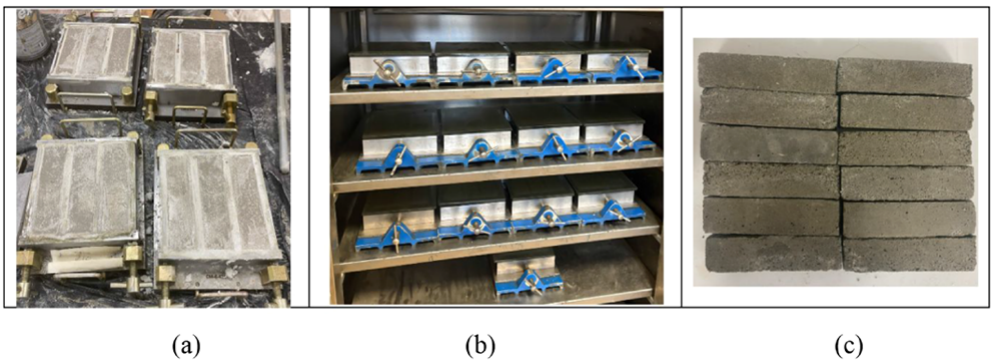
Preparation of 40 mm × 40 mm × 160 mm mortar specimens, (a) casting into molds, (b) curing, and (c) appearance after demolding.

### 3.4 Fresh-State Testing

The workability of the fresh state was assessed by measuring the flow diameter (FD) of the mortar mixtures using a small automatic flow table, in accordance with ASTM C1437.^23^ The test was conducted promptly after mixing to reduce the impact of time-dependent thixotropic activity. Fresh mortar was introduced into a truncated cone mold situated at the center of the micro flow table; subsequently, the cone was vertically elevated to permit the unimpeded flow of the mortar. The flow table was then engaged automatically to facilitate mortar dispersion.

Upon concluding the test, the spread diameter of the mortar was assessed in two orthogonal directions, and the mean of these measurements was recorded as the FD. This approach facilitated a reliable evaluation of mortar workability and ensured data consistency. The FD values were utilized to assess the impact of NT particle size and dosage on the rheological behavior of the mortar mixtures in their fresh state.

### 3.5 Physical Property Tests

WA and AP tests were conducted on hardened mortar specimens following ASTM C642.^24^ The tests assessed the impact of NT incorporation on pore-structure refinement and matrix densification.

### 3.6 Photocatalytic Tests

The photocatalytic self-cleaning performance of the mortar specimens was evaluated using a two-stage procedure consisting of dark adsorption followed by UV-A–induced photocatalytic discoloration. Representative images of the experimental stages, including methylene blue (MB) application and surface discoloration after irradiation, are provided in Figure 4. The photocatalytic performance of the mortar specimens was evaluated by monitoring the degradation of the methylene blue (MB) dye adsorbed on the specimen surface. Photocatalytic tests were conducted on prismatic mortar specimens (40 × 40 × 160 mm^3^), which were cured for 28 days prior to testing. Before the photocatalytic assessment, all specimens were oven-dried at 40 ± 2 °C until reaching constant mass and subsequently cooled to room temperature. The surfaces to be irradiated were gently cleaned to remove loose particles and surface contaminants. This preparation step is critical to ensure the repeatability and reliability of the photocatalytic measurements.

**Figure 4:**
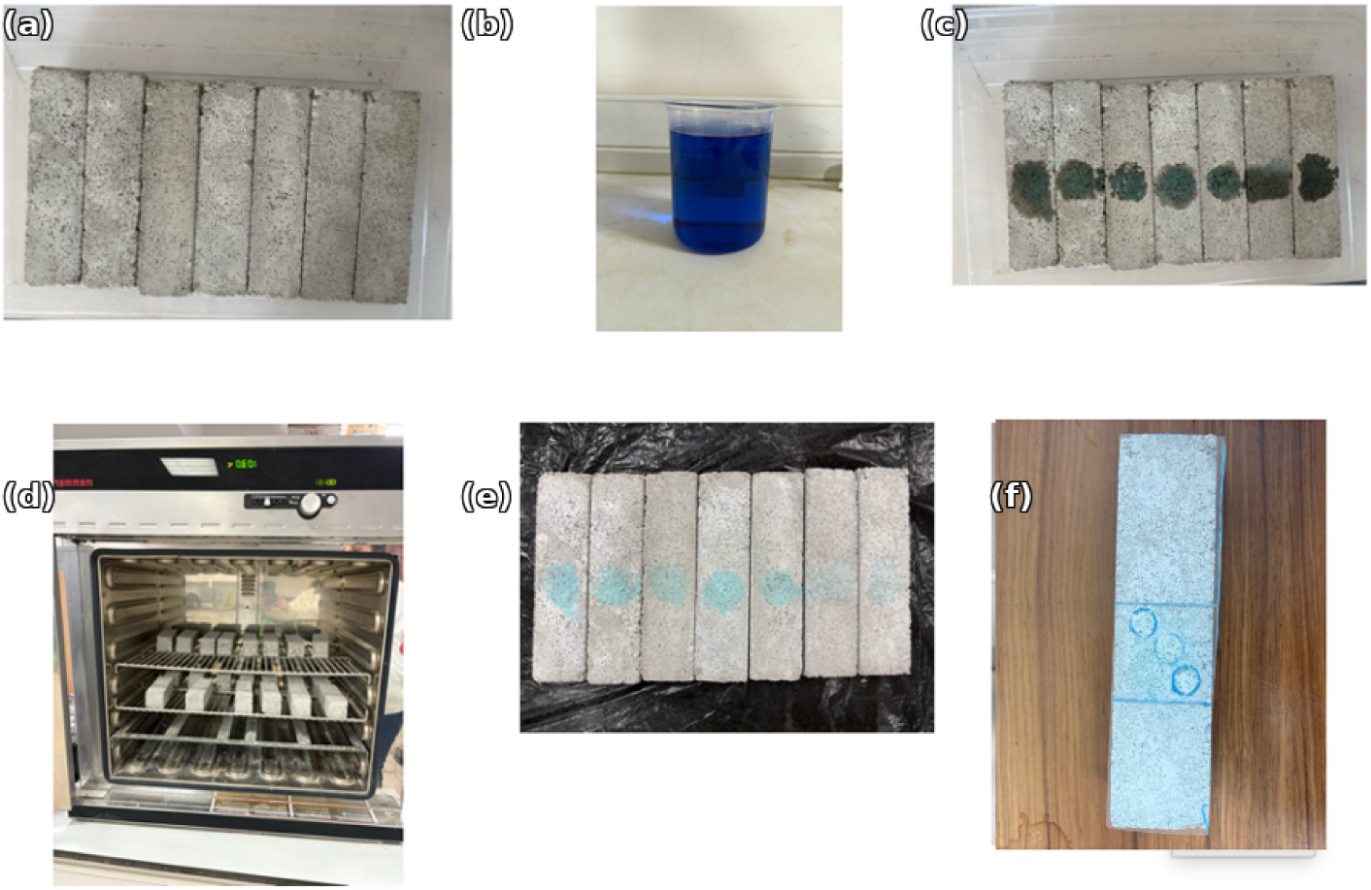
(a) Initial condition of specimens before testing, (b) methylene blue (MB) solution, (c) application of MB solution on specimen surfaces, (d) curing/conditioning process in the oven, (e) surface discoloration after photocatalytic reaction, and (f) detailed view of localized color change on the specimen surface.

For the staining procedure, MB was used according to ASTM C1378.^25^ The methylene blue (MB) solution was prepared by dissolving 1 g of methylene blue powder in 1 L of distilled water, then stirring at 500 rpm for 5 min to ensure complete dissolution and homogeneity. The surfaces of mortar specimens containing different NT combinations were uniformly coated with an aqueous MB solution. A volume of 0.4 mL of MB solution was applied to a defined surface area (4 cm × 4 cm) using a syringe. The coated specimens were then kept in the dark for 60 min to establish adsorption–desorption equilibrium before UV exposure.

Initial color measurements of the MB-coated specimens were performed using a portable spectrophotometer (PCE-CSM-2). Subsequently, the specimens were exposed to UV-A irradiation (365 nm, Philips PL-L 36W/09/4P) for 1, 2, and 4 h. The distance between the UV source and the specimen surface was fixed at 15 cm. To standardize the measurement locations, a template was placed on each specimen to ensure consistent color readings. For each specimen, three color measurements were taken, and the color difference (ΔE) was calculated accordingly. Surface color variations were quantified using the CIELAB color system, and the color difference (ΔE) was determined according to

1 $$\Delta E=\sqrt{\left[{\left(L_{t}-L_{0}\right)}^{2}+{\left(a_{t}-a_{0}\right)}^{2}+{\left(b_{t}-b_{0}\right)}^{2}\right]}$$

where L_0_, a_0_, and b_0_ correspond to the initial lightness and chromaticity coordinates after dark adsorption, while L_t_, a_t_, and b_t_ represent the respective values after UV irradiation time t. A higher ΔE value indicates greater surface discoloration and thus enhanced self-cleaning performance. The progressive reduction in surface color intensity was attributed to NT-induced, photocatalytic degradation of MB. Control specimens without NT were tested under identical conditions to provide a baseline for comparison.

After calculating the ΔE values of the mortar specimens, the surfaces coated with methylene blue (MB) solution were exposed to UV irradiation at predetermined time intervals. The color difference of each specimen at time t, ΔE­(t), was measured using a spectrophotometer. Based on these values, the MB degradation rate of each specimen was calculated as follows:

2 R=ΔE(t)−ΔE(0)/ΔE(0)×100

Here, ΔE(0) represents the initial color value of the mortar surface coated with methylene blue (MB) prior to UV exposure, whereas ΔE­(t) denotes the color value of the specimen surface after irradiation for a given exposure time t.

### 3.7 Mechanical Testing

FS tests were performed on prismatic mortar specimens measuring 40 mm × 40 mm × 160 mm, in accordance with ASTM C348.^26^ The flexural test was initially conducted utilizing a three-point bending configuration. After flexural testing, the two fractured halves from each prism were utilized for compressive strength testing.

Compressive strength tests were conducted in accordance with ASTM C349.^27^ This testing sequence ensured consistency in flexural and compressive strength measurements, facilitating the efficient use of test specimens. Mean values from various specimens were used to assess the mechanical performance of the mortar mixtures. The flexural and compressive strength tests were performed under load-controlled conditions at loading rates of 0.05 kN/s and 1.20 kN/s, respectively. Three specimens were tested for each assessment, and the results are reported as average values with their respective standard deviations.

### 3.8 Microstructural Analysis

Microstructural characterization was conducted using SEM on selected mortar specimens, which included the control mixture and representative NT-modified mixtures. SEM analysis was utilized to investigate the morphology of hydration products, the evolution of pore structure, and the dispersion state of NT particles in the cement matrix—microstructural observations supported and interpreted the trends observed in fresh-state behavior, physical properties, and mechanical performance. The experimental workflow is illustrated in Figure 5.

**Figure 5:**
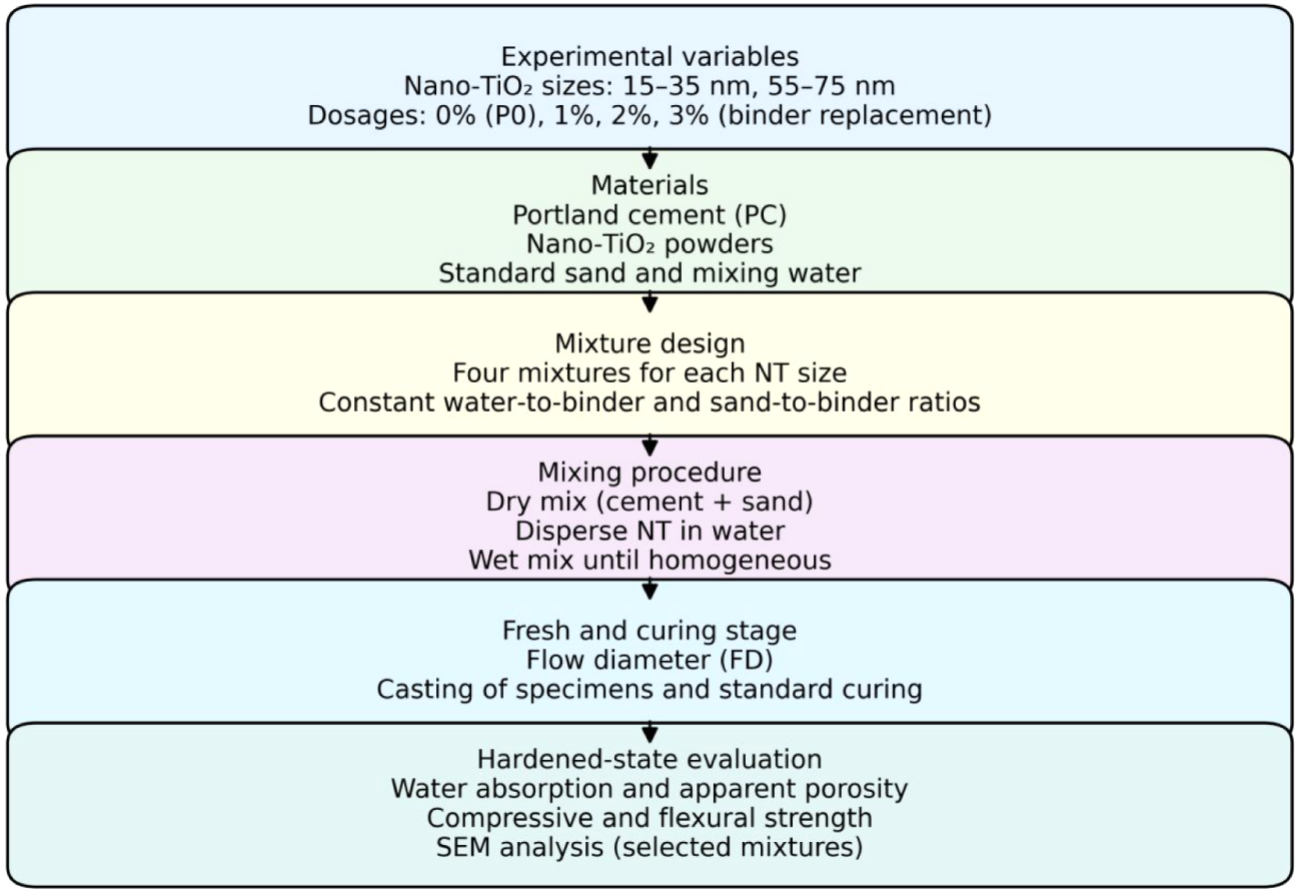
Schematic representation of the experimental procedure.

## 4 Results and Discussion

### 4.1 Ambient Conditions

#### 4.1.1 Flow Diameter (FD) of P0–P6 Mixes

The FD results for all mixtures indicate that the inclusion of NT decreases the workability of the mortars in the fresh state. The photographic appearances of the flow diameter (FD) for the P0, P3, and P6 mixtures are shown in Figure 6.

**Figure 6:**
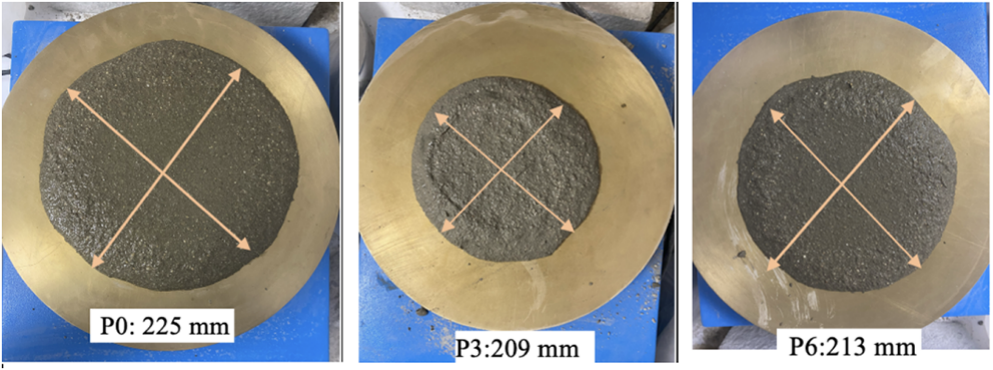
Photographic views of the flow diameter (FD) of P0, P3, and P6 mixtures.

The control mixture P0 had a flow diameter of 225 mm, indicating the highest flowability. In mixtures with 15–35 nm NT, the observed FD values were 221 mm, 216 mm, and 209 mm for P1 (1%), P2 (2%), and P3 (3%), respectively, indicating a systematic decrease in flow diameter with increasing NT content. For mixtures containing 55–75 nm NT, the FD values recorded were 223 mm for P4 (1%), 218 mm for P5 (2%), and 213 mm for P6 (3%). A comparable decreasing trend was noted; however, the reduction in flow diameter was consistently less significant than that observed for the 15–35 nm series at the same replacement levels. The quantitative results of the flow diameter (FD) are presented in Figure 7.

**Figure 7:**
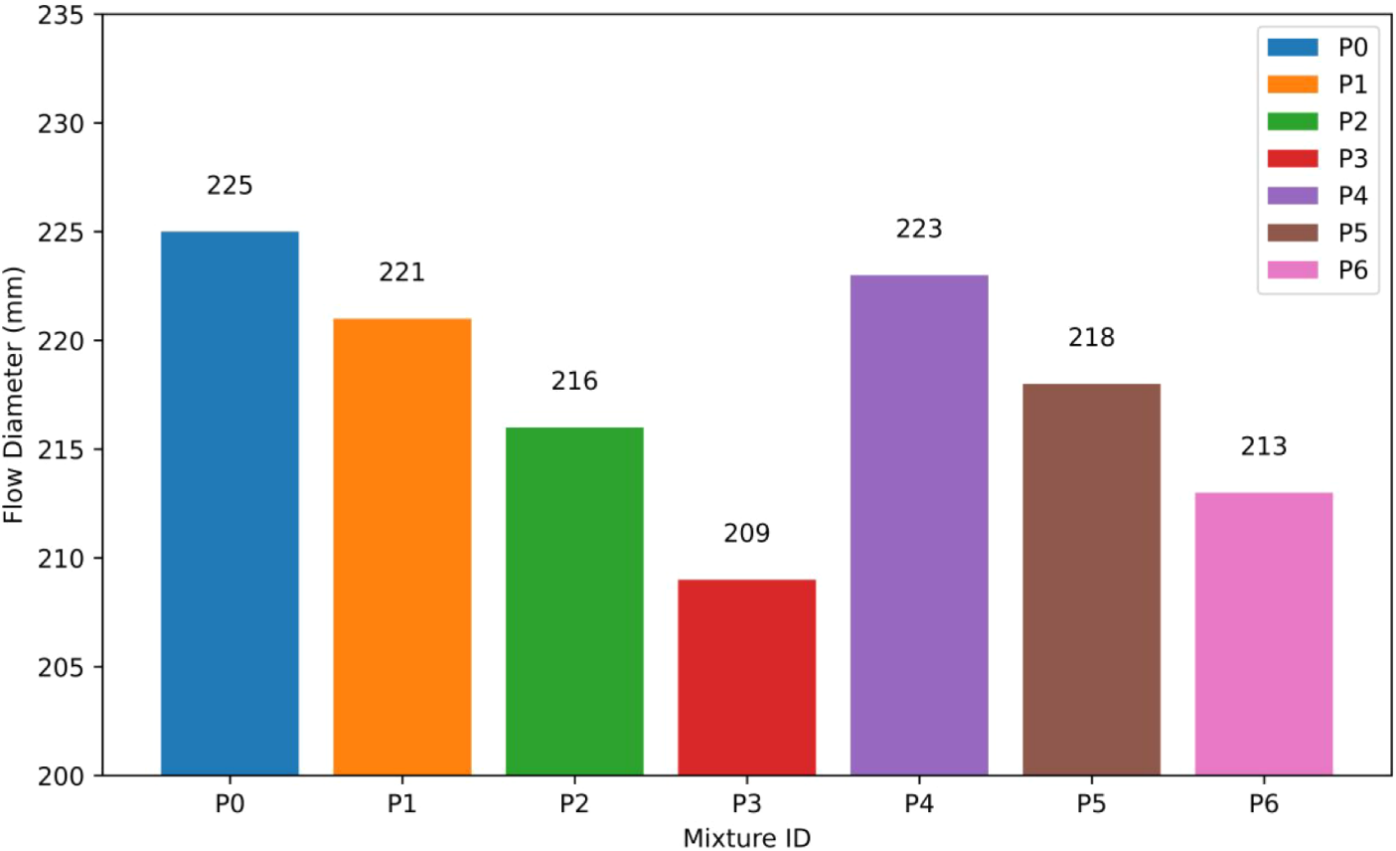
Flow diameter (FD) results of P0–P6 mixtures.

The observed reduction in FD is primarily due to the elevated SSA of NT particles, which significantly increases as particle size diminishes.^28^ Nanoparticles exhibit a significantly increased SSA that requires wetting during mixing, resulting in improved physical adsorption of mixing water onto the surfaces of the particles.^29^ The reduction in available free water for lubrication and particle rearrangement in fresh mortar leads to increased yield stress and decreased flowability.^30^ This mechanism accounts for the greater reduction in FD values noted in the 15–35 nm NT mixtures relative to those with 55–75 nm particles, which have a lower SSA and consequently require less water.^31^

Alongside SSA-related water adsorption, increased interparticle interactions play a role in the noted loss of workability. The elevated surface energy of NT particles enhances van der Waals^32^ and electrostatic attractions^33^ among particles, thereby augmenting internal friction within the fresh matrix. The effect intensifies as particle size decreases and NT dosage increases, thereby further limiting the mortar’s spread during the flow test. Furthermore, at elevated replacement levels, especially at 3% NT, there is an increased likelihood of particle agglomeration,^34^ resulting in the creation of rigid clusters that serve as mechanical barriers to flow and further diminish FD.

A secondary yet pertinent factor is the nucleation effect of NT on the initial stages of cement hydration.^35^ Nanoscale particles offer multiple nucleation sites for hydration products, thereby facilitating early structural development in the fresh mixture.^36^ While advantageous for microstructural refinement in later stages, the formation of this early-stage network may hinder particle mobility and lead to decreased FD. The interaction of water adsorption, interparticle attraction, agglomeration tendencies, and early hydration effects leads to a significant decrease in FD for finer nanoparticles and elevated replacement ratios.

#### 4.1.2 Water Absorption (WA) of P0–P6 Blends

The WA results show that adding NT significantly alters the way moisture moves through the mortars. The control mixture, P0, had a water absorption of 8.62%, which was used as the standard. When 15–35 nm NT was added to the mortars, the WA values dropped to 8.34%, 8.12%, and 8.18% for P1 (1%), P2 (2%), and P3 (3%), which is about 3.2%, 5.8%, and 5.1% lower than the control mixture. These results show that water resistance has improved significantly, with the largest drop occurring at 2% NT content. The water absorption (WA) results are presented in Figure 8.

**Figure 8:**
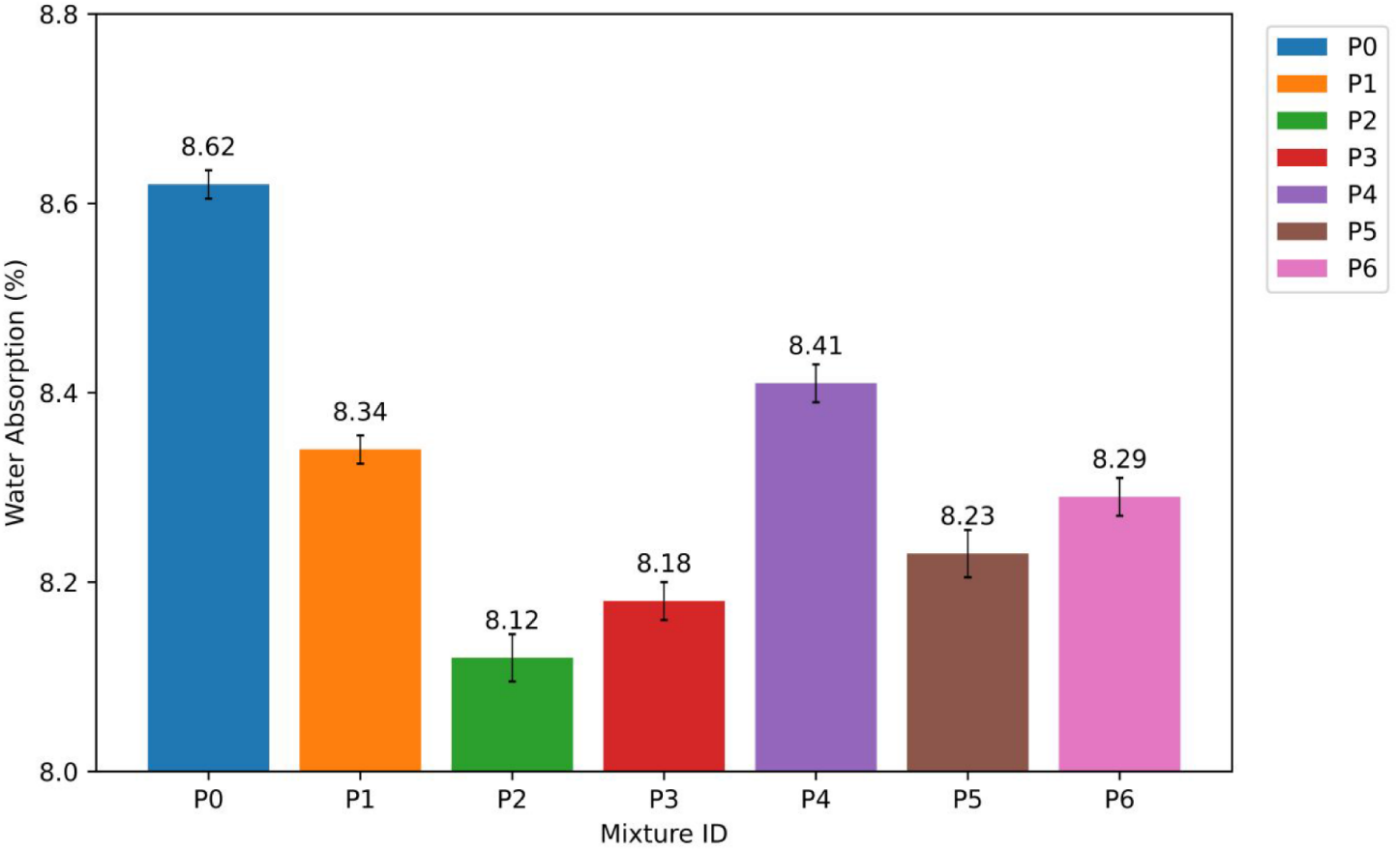
Water absorption (WA) results of P0–P6 mixtures.

In the same way, mixtures with 55–75 nm NT had WA values of 8.41% for P4 (1%), 8.23% for P5 (2%), and 8.29% for P6 (3%). These values were about 2.4%, 4.5%, and 3.8% lower than P0. All of the NT-modified mixtures had lower WA values than the control. However, the 15–35 nm series had a bigger drop in WA values than the 55–75 nm series at the same replacement levels.

The observed decreases in WA can be mainly linked to the pore-refining effect of NT particles.^37^ Because these particles are tiny, they fill in micro- and submicron voids in the cementitious matrix, which makes the capillary pore volume and pore connectivity smaller.^38^ This mechanism works best with 15–75 nm NT particles because they have a larger SSA and can get into smaller pore spaces better than 55–75 nm particles.^39^ This technique makes the microstructure denser and makes it harder for water to get in.

NT particles not only fill in gaps, but they also act as nucleation sites for hydration products.^40^ This helps make the calcium-silicate-hydrate (C–S–H) gel network more even and compact.^41^ The combined effects of pore filling and better hydration cause the significant decrease in WA observed at low to moderate levels of NT. But at the 3% replacement level, the small increase in WA compared to the best 2% mixtures suggests that nanoparticles are starting to clump together.^42^ These modifications could make the matrix less uniform in some places and lessen the positive effects of densification.

#### 4.1.3 Apparent Porosity of P0–P6 Blends

The AP results show that adding NT systematically improves the pore structure of the mortars. We used the AP value of 10.12% from the control mixture P0 as a reference. When mortars had 15–35 nm NT, the AP values decreased to 9.72%, 9.53%, and 9.61% for P1 (1%), P2 (2%), and P3 (3%), respectively. This was a drop of about 3.95%, 5.83%, and 5.04%, respectively, from P0. At a replacement level of 2% NT, the AP was at its lowest. The apparent porosity (AP) results are illustrated in Figure 9.

**Figure 9:**
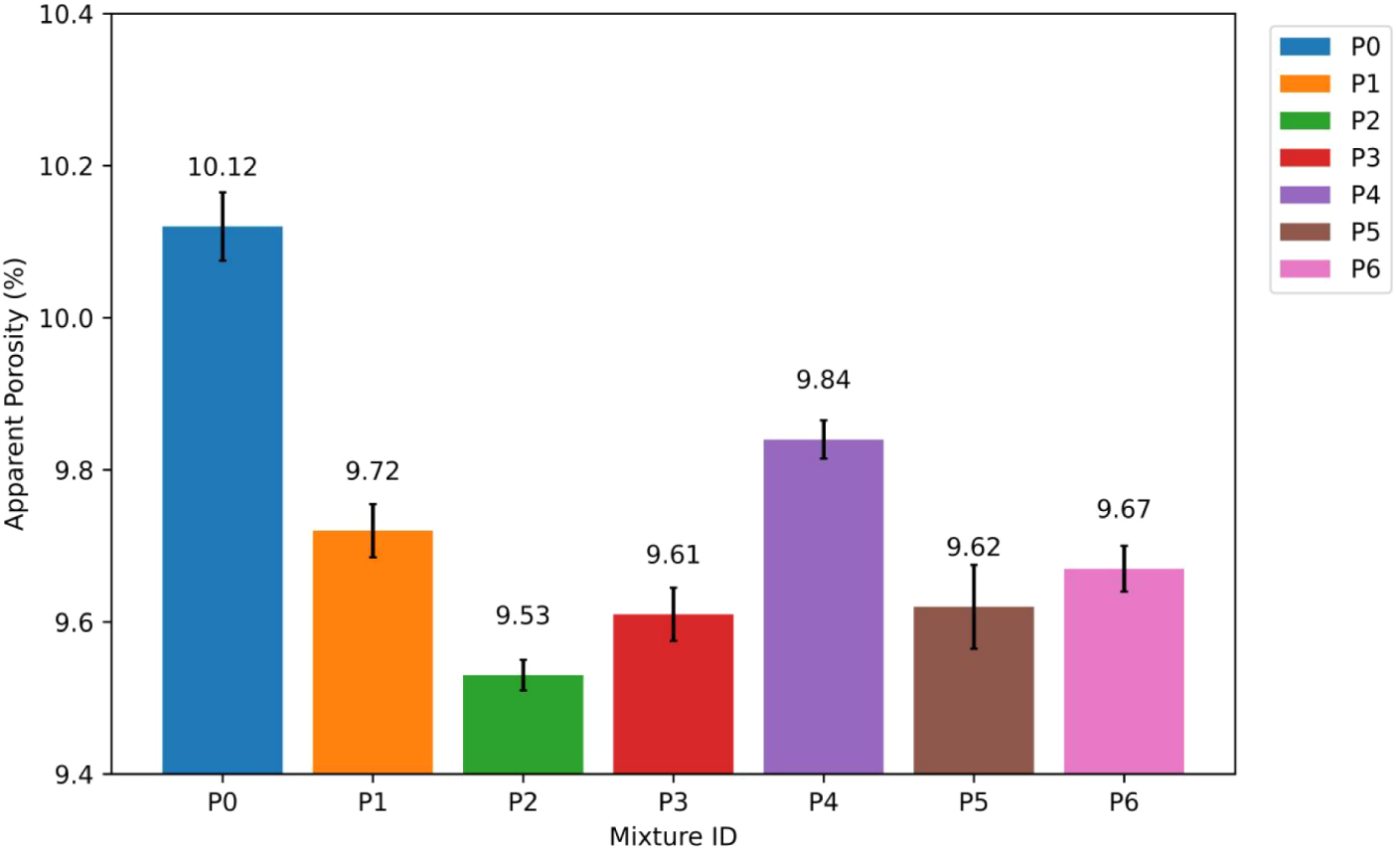
Apparent porosity (AP) results of P0–P6 mixtures.

In the same way, mixtures with 55–75 nm NT had AP values of 9.84% for P4 (1%), 9.62% for P5 (2%), and 9.67% for P6 (3%). These values were about 2.77%, 4.94%, and 4.45% lower than the control mixture, respectively. All NT-modified mortars had lower AP values than P0, but the difference was always bigger in the 15–35 nm NT series than in the 55–75 nm NT series at the same replacement levels.

The decrease in AP is mostly due to the microfilling effect of NT particles, which lets them fill in small capillary pores and voids in the cementitious matrix.^43^ Because they are smaller and have a larger SSA, 15–35 nm NT particles can get into microscale pores better than 55–75 nm NT particles. This process makes the packing density higher and the pore connectivity lower. This mechanism elucidates the enhanced AP reduction noted in the P1–P3 mixtures.

NT particles not only act as fillers, but they also serve as nucleation sites for hydration products, which helps create a more uniform and compact C–S–H network.^44^ The faster and more even precipitation of hydration products helps refine the pores even more and explains why the AP goes down when the NT content is low to moderate. At the 3% NT replacement level, the small rise in AP compared to the best 2% mixtures suggests that nanoparticles are starting to clump together.^45^ Such behavior could make the matrix less uniform in some places and lessen the positive effects of densification.

#### 4.1.4 Compressive Strength of P0–P6 Blends

The CS results at 3, 7, and 28 days show that adding nano-TiO_2_ (NT) has a significant effect on both the early strength growth and the compressive performance after 28 days. This phenomenon occurs due to particle size and the amount of NT used. After 3 days, the control mixture P0 had a CS value of 23.51 MPa; after 7 days, 33.76 MPa; and after 28 days, 44.57 MPa. The percentages were based on these figures. The compressive strength (CS) results are shown in Figure 10.

**Figure 10:**
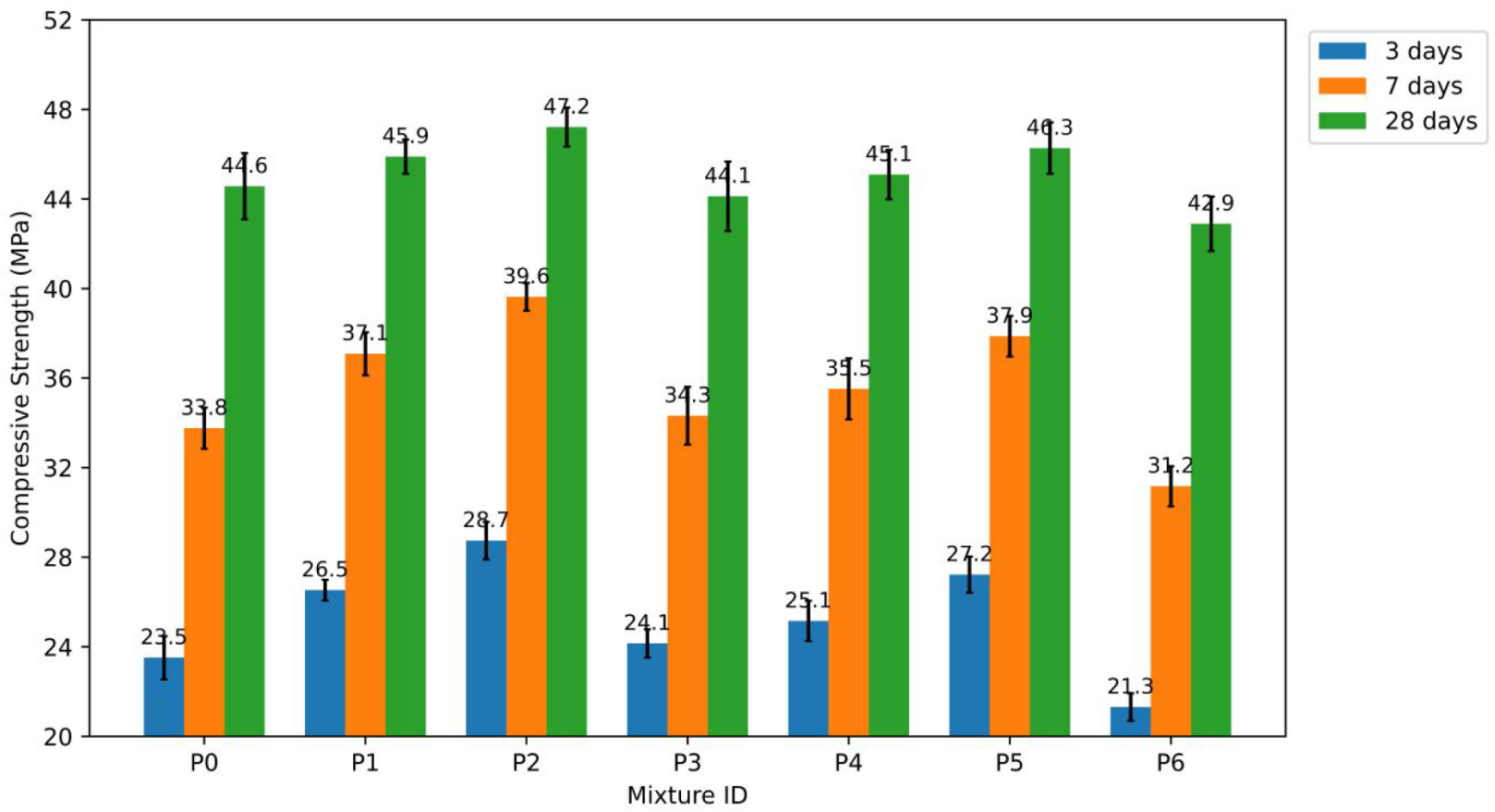
Compressive strength (CS) results of P0–P6 mixtures.

The CS improved at low and medium NT levels for combinations with 15–35 nm NT (P1–P3). At 1% NT (P1), the CS increased by about 12.8% after 3 days, 9.9% after 7 days, and 3.0% after 28 days compared to P0. The CS values were highest at all curing ages when the NT concentration was raised to 2% (P2). Compared with the control combination, they increased by approximately 22.2% at 3 days, 17.4% at 7 days, and 5.9% at 28 days. These findings indicate that incorporating an appropriate amount of fine NT can expedite the process and sustain strength gains.^46^

When the NT concentration was increased to 3% (P3), the positive effect weakened. CS at 3 and 7 days indicated minor increases of about 2.7% and 1.7%, respectively, as compared to P0. However, CS declined by roughly 1.0% after 28 days. This tendency suggests that having too high NT content makes it tougher to build strength, even though it might help with nucleation in other ways.^47^

The same thing happened with mixtures that had 55–75 nm NT (P4–P6), but the strength increase was usually less than with the 15–35 nm series. After 3 days, CS was approximately 7.0% higher than P0; after 7 days, approximately 5.2% higher; and after 28 days, approximately 1.2% higher. At 2% NT (P5), CS was around 15.8% better after 3 days, 12.2% better after 7 days, and 3.8% better after 28 days than the control combo. This result was a step in the right direction. CS levels were lower than P0 levels at 3% NT (P6). This indicates that they declined by approximately 9.4% after 3 days, 7.7% after 7 days, and 3.8% after 28 days.

The observed patterns can be ascribed to the synergistic effects of NT particle size and dosage on hydration kinetics and microstructural development.^48^ The 15–35 nm NT series often showed bigger gains in CS at a younger age than the 55–75 nm NT series. The reason for this difference is that smaller particles have a larger SSA and are better at generating nuclei.^49^ These characteristics accelerate hydration kinetics and promote the formation of a denser and more interconnected network of hydration products, particularly at early curing ages.^50^ At low NT levels, like 2%, these actions improve the packing density and make the pore structure more polished. This increases CS at every age of curing.^51^ However, when NT content is higher, the need for more water and the tendency of nanoparticles to aggregate can lead to less uniform distribution and hydration. These factors can impede or even reverse strength gains.^52^

#### 4.1.5 Flexural Strength of P0–P6 Blends

The findings at 3, 7, and 28 days indicate that the incorporation of NT significantly alters flexural performance during the initial days. After 28 days, it remains ineffective. The FS values for the control mixture P0 were 2.51 MPa at 3 days, 3.47 MPa at 7 days, and 4.21 MPa at 28 days. The numbers served as the foundation for percentage comparisons. The flexural strength (FS) results are presented in Figure 11.

**Figure 11:**
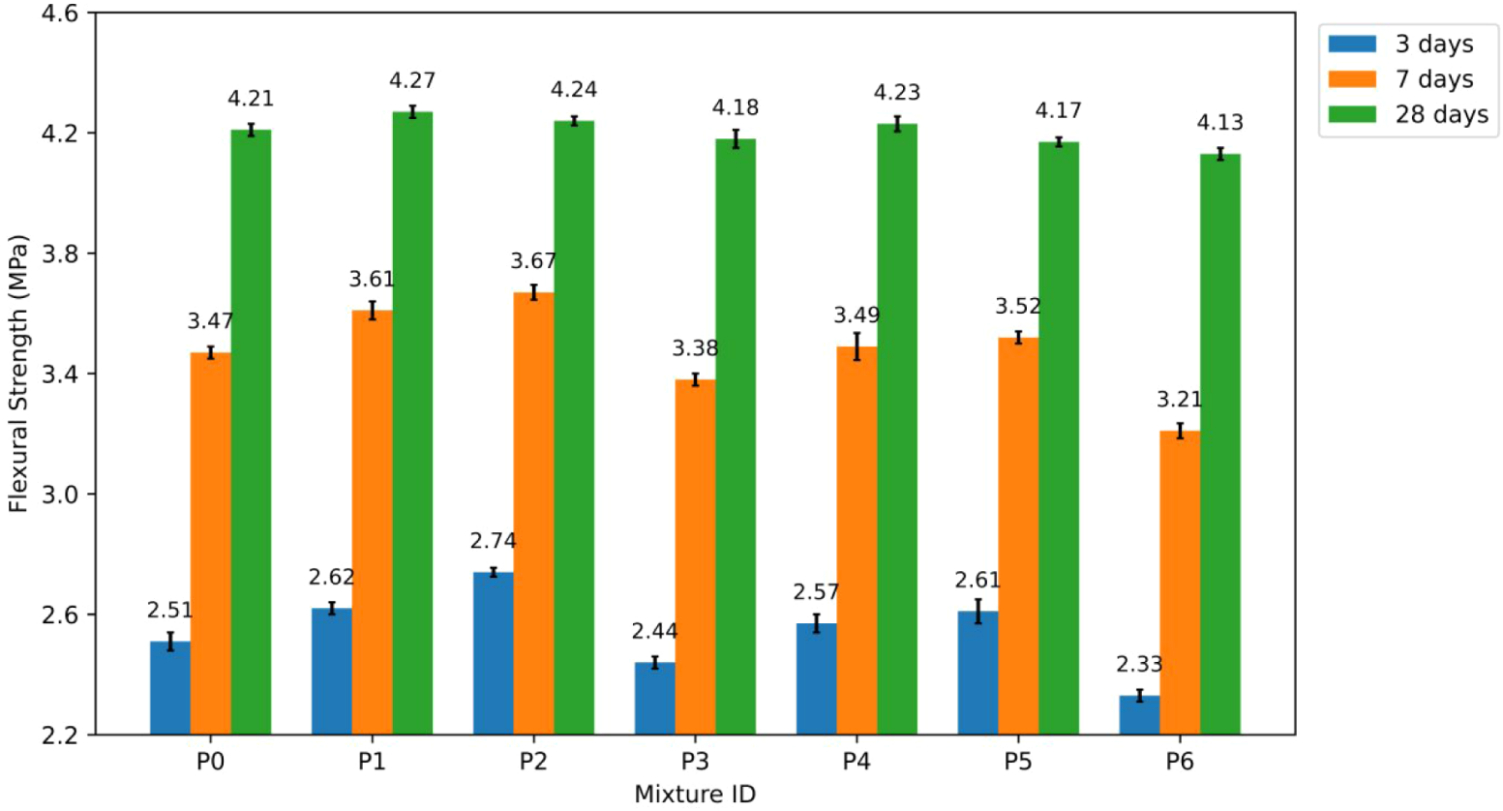
Flexural strength (FS) results of P0–P6 mixtures.

In scenarios where the NT proportion was low or moderate, FS outperformed P0 in combinations involving 15–35 nm NT (P1–P3). After 3 days, the FS was 4.0%; after 7 days, it was 1.4%; and after 28 days, FS was approximately 4.4% higher than the control combination at 1% NT (P1). The early-age FS values peaked at an NT content of 2% (P2). The level was 9.2% higher at 3 days and 5.8% higher at 7 days than at P0. At 28 days, the FS value was marginally elevated compared to the control value, by approximately 0.7%. Increasing the NT concentration to 3% (P3) resulted in a decrease in FS levels across all age groups compared to P0. Reductions were approximately 2.8% after 3 days, 2.6% after 7 days, and 0.7% after 28 days. This pattern indicates that excessive NT hinders FS enhancement.

Similar patterns were observed in combinations with 55–75 nm NT (P4–P6), although these were generally weaker. At an NT level of 1% (P4), FS exhibited increases of approximately 2.4% at 3 days, 0.6% at 7 days, and 0.5% at 28 days relative to P0. At 2% NT (P5), FS increased by approximately 4.0% after 3 days and 1.4% after 7 days. The 28-day FS value was marginally lower than the control value, indicating a decrease of approximately 1.0%. At 3% NT (P6), FS values were generally lower than P0 values. FS values decreased by approximately 7.2% after 3 days, 7.5% after 7 days, and 1.9% after 28 days.

The observed FS trends suggest that NT primarily affects early-age flexural performance, especially when utilizing finer particles and moderate replacement levels.^53^ The 15–35 nm NT combinations typically enhanced FS at an earlier age than the 55–75 nm NT mixtures.^54^ The observed effect arises from the larger SSA of smaller particles, which enhances their nucleating efficiency.^55^ This phenomenon increases matrix density and mitigates the early formation of microcracks.^56^ The marginal increase in 28-day FS indicates that NT alone is insufficient to enhance flexural behavior without fiber reinforcement. This phenomenon occurs because crack-bridging mechanisms significantly influence flexural strength beyond merely increasing the matrix density.^57^

#### 4.1.6 Photocatalytic Self-Cleaning Performance of NT-Modified Mortars

The photocatalytic performance of the mortars was evaluated using methylene blue (MB) degradation, measured by ΔE color variation. The experimental ΔE values together with their error bars are presented in Figure 12, while the temporal evolution trends are more clearly illustrated in Figure 13.

**Figure 12:**
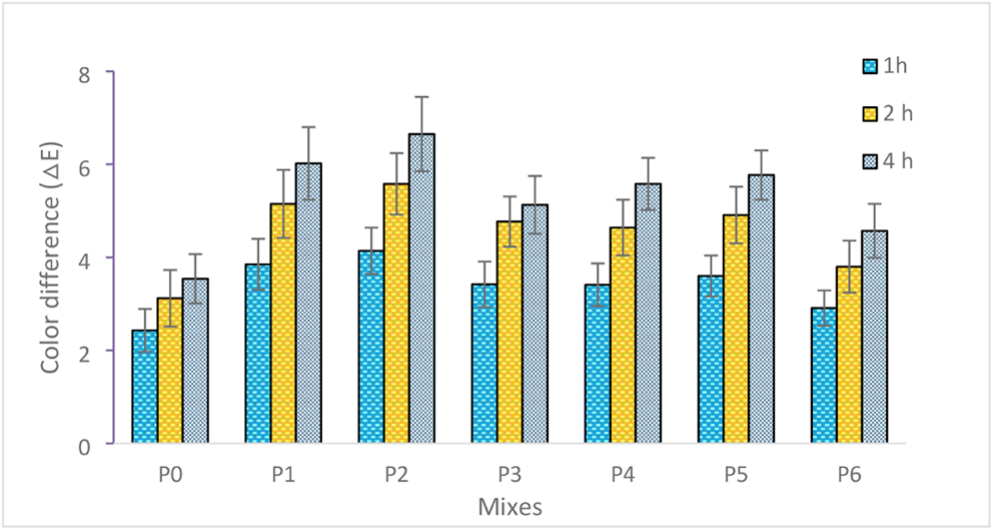
Photocatalytic self-cleaning performance of mortar mixtures incorporating different nano-TiO_2_ particle sizes and replacement ratios, expressed as color difference (ΔE) after 1, 2, and 4 h of UV-A irradiation.

**Figure 13:**
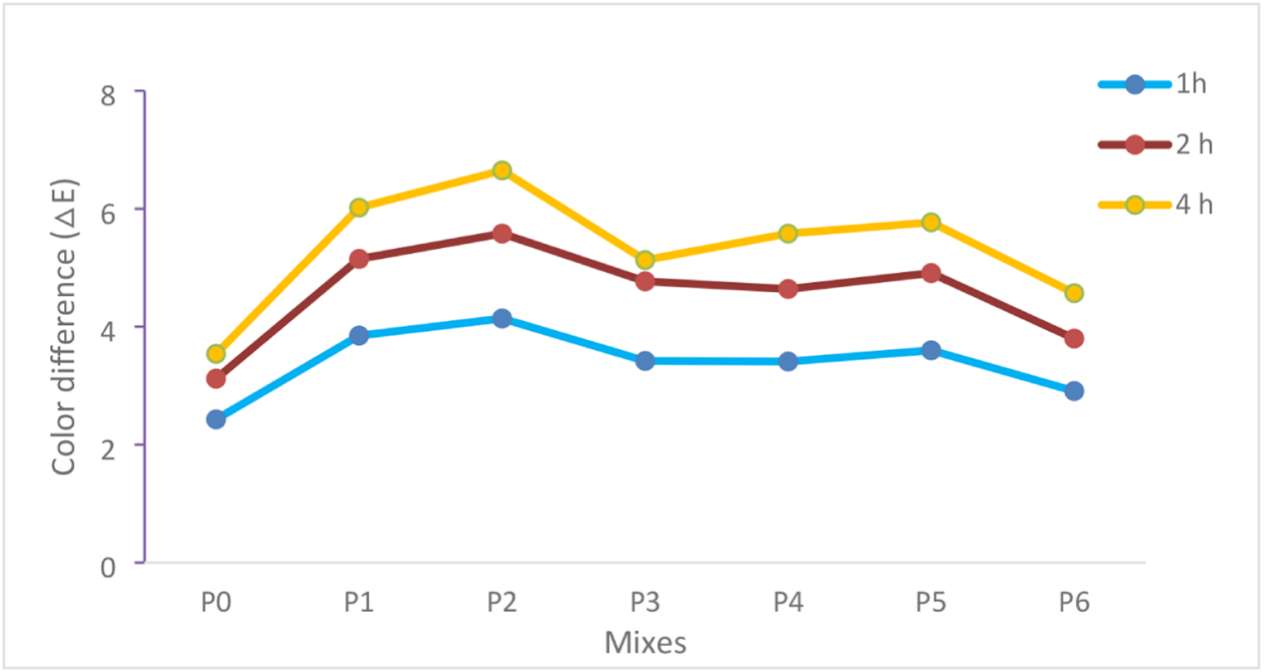
Time-dependent photocatalytic response of nano-TiO_2_-modified mortar mixtures, expressed as ΔE under UV-A irradiation.

As shown in Figure 12, the control specimen P0 (0% nano-TiO_2_) exhibited limited color variation, with ΔE values of 2.43, 3.12, and 3.54 after 1, 2, and 4 h of UV exposure, respectively. This gradual increase indicates minor photobleaching effects rather than true photocatalytic oxidation. In contrast, all nano-TiO_2_-modified mixtures (P1–P6) demonstrated significantly higher ΔE values at each exposure duration, confirming the active role of TiO_2_ in radical-driven degradation reactions.

For the mortars incorporating 15–35 nm nano-TiO_2_, P1 (1%) reached ΔE values of 3.85, 5.15, and 6.48 at 1, 2, and 4 h, respectively. The mixture P2 (2%) exhibited the highest photocatalytic efficiency among all specimens, with ΔE values of 4.14, 5.58, and 6.76. Compared to the control specimen at 4 h (ΔE = 3.54), P2 achieved approximately a 91% higher color variation, confirming enhanced electron–hole generation and reactive oxygen species (•OH and •O_2_^–^) formation.^58^ However, increasing the replacement ratio to 3% (P3) resulted in slightly lower ΔE values (3.42, 4.77, and 6.23), suggesting that excessive nano-TiO_2_ content may promote particle agglomeration,^59^ reduce effective surface area,^60^ and increase the probability of charge recombination.^61^ This behavior indicates the presence of an optimum dosage level rather than a linear relationship between nano-TiO_2_ content and photocatalytic performance.^62^

A similar trend was observed for the 55–75 nm particle group. P4 (1%) and P5 (2%) exhibited ΔE values up to 6.29 and 6.12 at 4 h, respectively, whereas P6 (3%) showed a comparatively lower value of 5.88. As illustrated in Figure 13, the peak photocatalytic response consistently occurred at 2% replacement for both particle size groups, while further increase to 3% resulted in performance reduction. Additionally, at equivalent replacement levels, the finer particle group (15–35 nm) consistently outperformed the coarser group (55–75 nm), confirming the critical influence of specific surface area on photocatalytic efficiency.

These findings are consistent with the literature. Coffetti et al.^63^ reported that nano-TiO_2_-modified mortars exhibited noticeable variations in color coordinates, particularly in the b* parameter, with changes ranging between approximately 10% and 40% after UV exposure, indicating enhanced photocatalytic degradation of MB. Similarly, Tyukavkina et al.^64^ observed that ΔE values increased progressively with UV exposure time in cement pastes containing 1% nano TiO_2_–SiO_2_, demonstrating sustained surface oxidation activity. In comparison, the ΔE values obtained in the present study—reaching up to 6.76 after 4 h—indicate a strong photocatalytic response and further highlight the importance of optimizing both particle size and dosage.

The progressive increase in ΔE values with exposure time across all nano-TiO_2_-modified mixtures (Figure 13) confirms sustained photocatalytic activity under UV irradiation. Overall, the combined interpretation of Figures 12 and 13 demonstrates that nano-TiO_2_ incorporation significantly enhances photocatalytic functionality in cement mortars, with optimum performance achieved at 2% replacement and a smaller particle size (15–35 nm), where dispersion efficiency, active surface area, and radical generation are optimally balanced.

#### 4.1.7 SEM

SEM analysis was conducted to elucidate the influence of nanotitanium dioxide (NT) incorporation on the microstructural characteristics of cement mortars, with particular emphasis on matrix densification, pore refinement, and the formation and spatial distribution of hydration products. Representative SEM micrographs of the P0, P3, and P6 mixtures are presented in Figure 14, while ImageJ-assisted quantitative threshold segmentation highlighting surface pore/void regions is shown in Figure 14. In addition to qualitative visual assessment, ImageJ-based 2D segmentation enabled comparative evaluation of relative pore area fraction and pore dispersion. The combined SEM observations and quantitative surface-void visualization provide complementary insight into matrix compactness and pore refinement trends.

**Figure 14:**
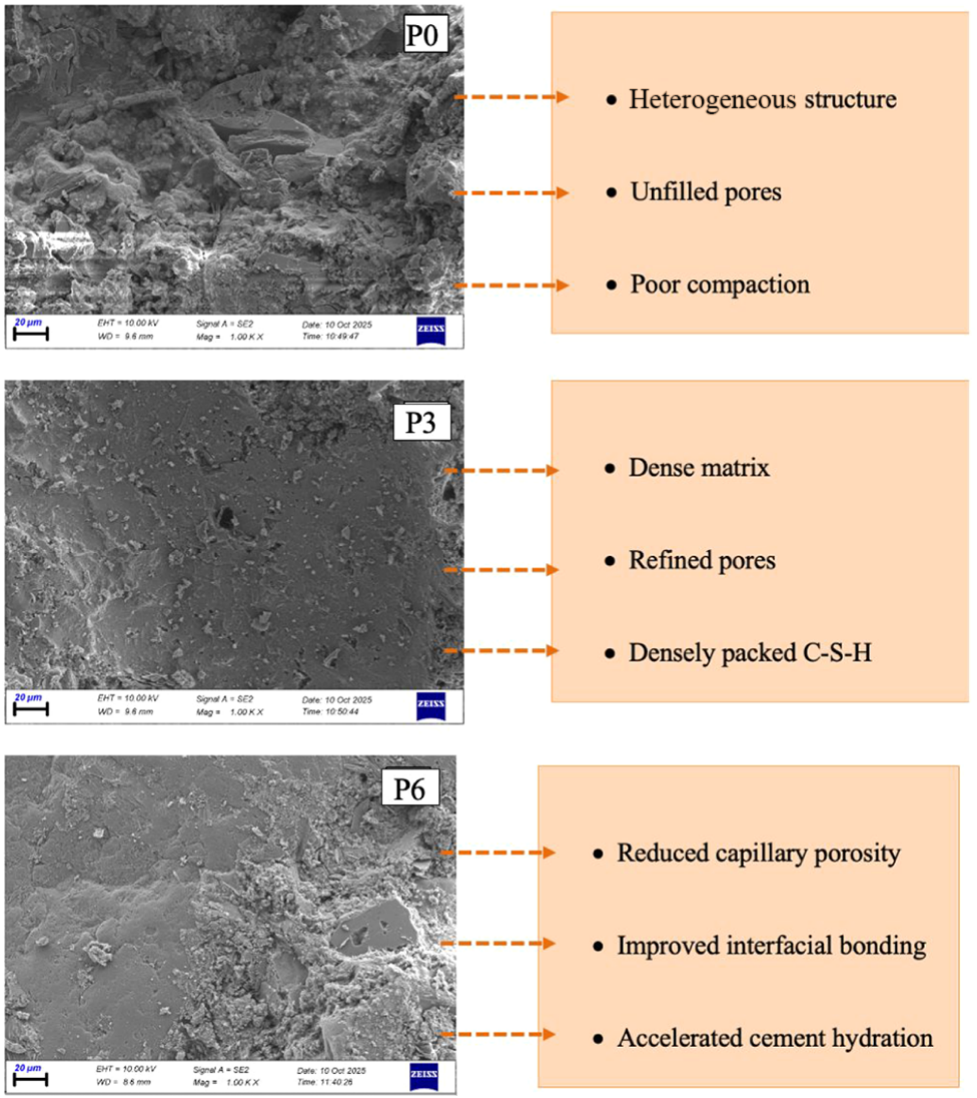
SEM images of P0, P3, and P6 mixtures.

The control mortar (P0) exhibits a heterogeneous and comparatively porous microstructure. SEM observations reveal loosely arranged hydration products, distinct capillary pores,^65^ and an irregular distribution of C–S–H gel,^66^ with clear boundaries between hydrated and unhydrated cement grains, indicating limited matrix continuity. Partially open interstitial spaces further increase pore connectivity.^67^ ImageJ-assisted segmentation (Figure 14) confirms a larger proportion of interconnected surface void regions in P0, supporting the higher WA and AP values and the relatively lower early-age mechanical performance of the control mixture. The incorporation of 15–35 nm NT leads to pronounced microstructural refinement (Figure 14), characterized by a denser, more homogeneous cement matrix^68^ and finer, more uniformly distributed hydration products.^69^ Owing to their elevated specific surface area (SSA), these nanoparticles act effectively as microfillers, occupying nano- and microscale voids within the matrix,^70^ while simultaneously serving as nucleation sites that promote C–S–H precipitation and the development of a more continuous hydration network.^71^

The combined effects of filler and nucleation substantially reduce pore size and connectivity, thereby enhancing matrix densification.^72^ This refinement is supported visually by the reduced, more dispersed surface-void regions highlighted in Figure 15. At higher NT contents, however, localized particle agglomeration becomes evident, disrupting microstructural uniformity and diminishing pore-refinement efficiency.^73^

**Figure 15:**
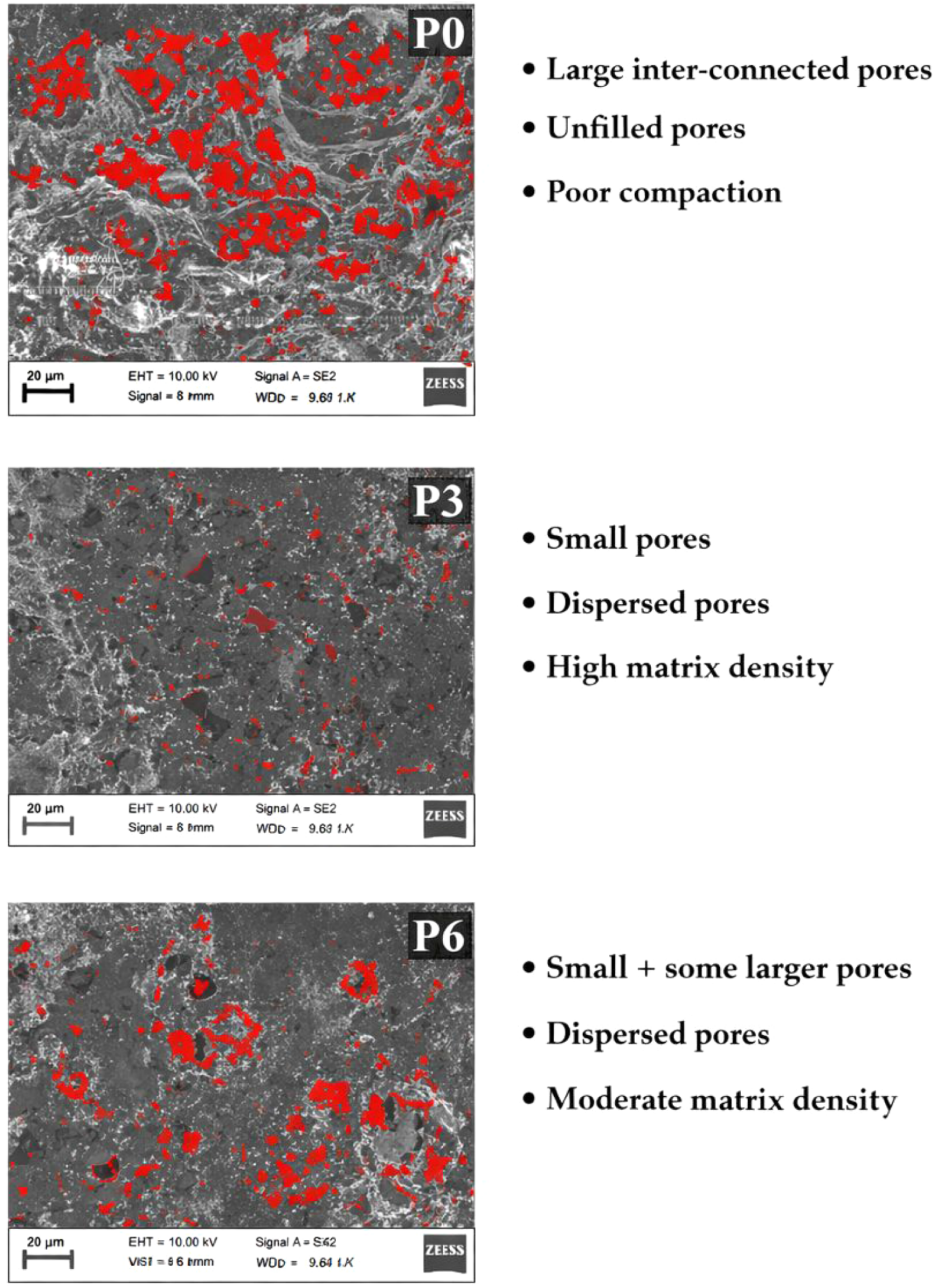
ImageJ-based threshold segmentation of SEM micrographs (1000×) for P0, P3, and P6, showing surface pore regions highlighted in red.

Mortars incorporating 55–75 nm NT also exhibit improved compactness relative to P0 (Figures 13 and 14); however, the degree of refinement is generally less pronounced than that achieved with 15–35 nm NT. The larger particle size and lower SSA of 55–75 nm NT limit penetration into finer pores and reduce nucleation efficiency.^74^ Although partial pore filling and enhanced C–S–H distribution are observed, fine-scale pore refinement remains constrained.^75^ Moreover, increasing the NT dosage promotes particle clustering, resulting in greater microstructural heterogeneity, as evidenced by localized void accumulation in Figure 14.^76^

## 5 Cost Estimation and Performance Evaluation of NT-Modified Mortars

This section evaluates the economic implications of incorporating nanotitanium dioxide (NT) into cement mortars by linking variations in binder-material costs to the development of compressive strength.^77^ Although NT is known to enhance mechanical performance and microstructural refinement,^78^ performance improvements alone are insufficient to justify practical implementation without economic feasibility.^79^ Therefore, a cost-performance assessment was conducted to determine whether the mechanical benefits from NT incorporation offset the associated increase in material cost.^80^

NT was used as a partial cement replacement at 1–3% by weight, and two NT particle size ranges (15–35 nm and 55–75 nm) were investigated separately (i.e., not in a combined or hybrid form). The total binder content was kept constant at 450 g, and the water-to-binder ratio (w/b), defined as the mass ratio of mixing water to total binder, was fixed at 0.50. Because sand, water, and admixture contents were unchanged across mixtures, the cost analysis was limited to binder materials only (cement + NT), thereby ensuring a direct evaluation of the economic impact of NT substitution.

Unit prices were derived from the authors’ procurement data: ordinary Portland cement cost 635 Turkish Lira (TL) per 25 kg (25.4 TL/kg). NT prices were substantially higher, amounting to 5400 TL per 100 g for the 15–35 nm particles (54,000 TL/kg) and 4900 TL per 100 g for the 55–75 nm particles (49,000 TL/kg). Since these values correspond to laboratory-scale quantities, the calculated cost increments should be interpreted as an upper-bound estimation of the NT-related economic impact, unless reduced bulk-purchasing prices are available.

The relative cost increase (RCI) was defined as the percentage increase in binder-material cost of an NT-modified mixture relative to the control mixture without NT and was calculated using eq 3. This approach enables a balanced assessment of the economic burden introduced by NT incorporation in relation to the achieved compressive strength enhancement, providing a practical perspective on the applicability of NT-modified cement composites in engineering practice.

3 $$\mathrm{RCI}\;\left(\%\right)=\left[\left(C_{\mathrm{NT}}-C_{0}\right)/C_{0}\right]\times 100$$

In eq 4, C_NT_ (TL) is the total binder-material cost of the NT-modified mixture, and C_0_ (TL) is the total binder-material cost of the control mixture (P0). Mechanical benefit was expressed as compressive strength gain (CS Gain), calculated using eq 3:

4 $$\mathrm{CS Gain}\;\left(\%\right)=\left[\left({\mathrm{CS}}_{\mathrm{NT}}-{\mathrm{CS}}_{0}\right)/{\mathrm{CS}}_{0}\right]\times 100$$

Here, CS_NT_ (MPa) and CS_0_ (MPa) are the compressive strengths of NT-modified and control mixtures, respectively, measured at the same curing age. To relate economic input to mechanical benefit, a cost-performance index (CPI) was introduced as eq 5:

5 CPI=CS Gain(%)/RCI(%)

Higher CPI values indicate more efficient use of NT, whereas negative CPI values reflect strength loss combined with cost increases (diminishing returns). Table 1 summarizes the computed indicators. Using the above laboratory-scale NT prices, RCI values are very high even at low NT contents, indicating that economic feasibility is highly sensitive to NT procurement prices and purchase quantities. Therefore, the optimum NT dosage from a cost perspective should be interpreted cautiously and may shift substantially if bulk NT prices are used. The calculated binder-material costs, relative cost increase (RCI), compressive strength (CS), compressive strength gain, and cost-performance index (CPI) values for all mortar mixtures are summarized in Table 4. As indicated in Table 4, the incorporation of nanotitanium dioxide (NT) results in a substantial increase in binder-material cost even at low replacement levels, reflecting the significantly higher unit price of NT compared to ordinary Portland cement. This cost escalation becomes more pronounced as NT content increases and is slightly higher for finer NT particle sizes due to their higher procurement costs.

**Table 4:** Cost-Performance Indicators of NT-Modified Mortars

Mix ID	NT size (nm)	NT (%)	Binder cost, C (TL)	RCI (%)	CS (MPa)	CS Gain (%)	CPI
P0	–	0	11.43	0.0	44.57	0.0	0.0
P1	15–35	1	254.32	2125.0	45.89	2.96	0.001394
P2	15–35	2	497.2	4250.0	47.21	5.92	0.001394
P3	15–35	3	740.09	6375.0	44.12	–1.01	–0.000158
P4	55–75	1	231.82	1928.1	45.09	1.17	0.000605
P5	55–75	2	452.2	3856.3	46.27	3.81	0.000989
P6	55–75	3	672.59	5784.4	42.89	–3.77	–0.000652

The variation of relative cost increase (RCI) as a function of NT content for the two NT particle size ranges is illustrated in Figure 16. As shown in Figure 16, RCI increases almost linearly with increasing NT dosage for both particle sizes, with higher RCI values consistently observed for the finer NT. This trend confirms that NT dosage is the dominant factor governing cost escalation under laboratory-scale procurement conditions.

**Figure 16:**
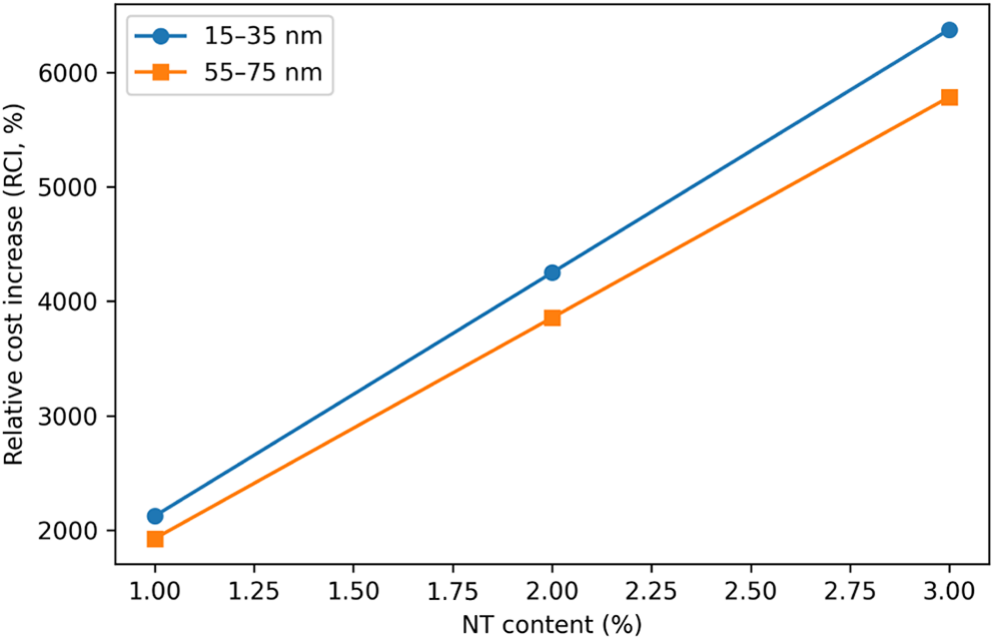
Relative cost increase (RCI) as a function of NT content for the two NT particle size ranges.

To evaluate the economic efficiency of NT incorporation, the relationship between the cost-performance index (CPI) and NT content is presented in Figure 16. As shown in Figure 17, mortar mixtures incorporating 1–2% NT exhibit the highest CPI values for both particle size ranges, indicating a more favorable balance between mechanical performance enhancement and economic input. In contrast, at the highest NT replacement level (3%), CPI values decrease sharply and become negative for some mixtures, demonstrating diminishing mechanical returns despite a substantial increase in material cost.

**Figure 17:**
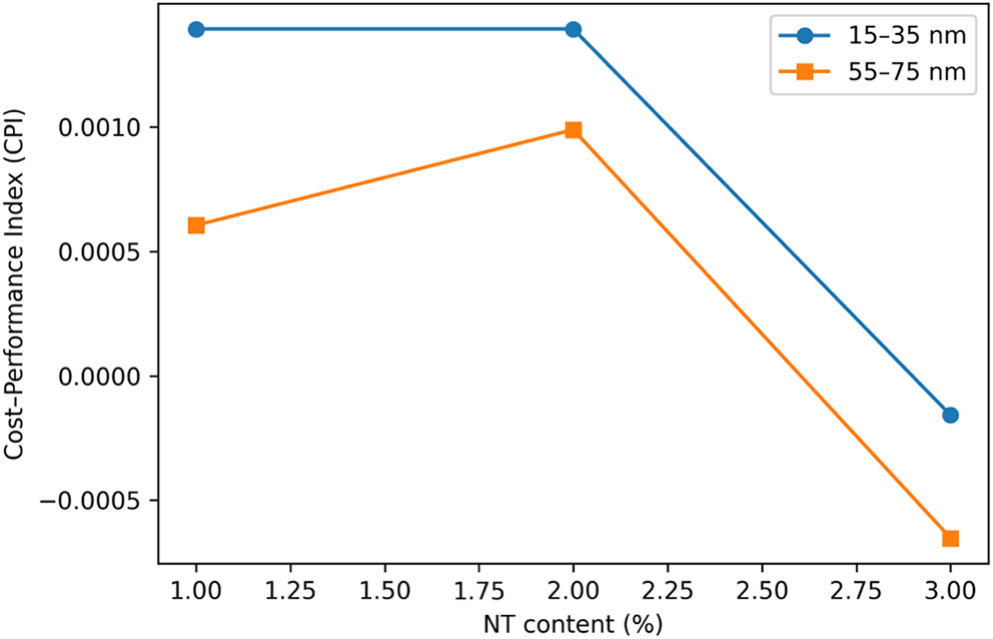
Cost-performance index (CPI) versus NT content for the two NT particle size ranges.

## 6 Conclusions

This research systematically assessed the impact of incorporating NT on the fresh, physical, mechanical, and microstructural properties of cement mortars using a replacement-based approach. The results indicate that NT particle size and dosage significantly influence performance across various scales and curing ages.

- NT addition reduced the FD of the mortars. This reduction was more pronounced for the 15–35 nm particles due to their higher specific surface area and associated water demand compared to the 55–75 nm particles.
- WA and AP values decreased with NT incorporation due to the filler effect of nanosized particles, which effectively filled micropores and reduced capillary connectivity. This reduction was more pronounced for the 15–35 nm particles because their smaller size enabled more efficient pore filling than the 55–75 nm particles.
- Due to particle agglomeration, mortars containing 3% NT exhibited slightly higher water absorption and apparent porosity values compared to the 1% and 2% NT mixtures. This agglomeration effect reduced the efficiency of pore refinement at higher NT dosages.
- The increase in CS is attributed to the NT filler effect, which fills microvoids and densifies the cement matrix.
- NT also acted as effective nucleation sites for hydration products, accelerating hydration and contributing more significantly to early-age strength (3 and 7 days) than to 28-day strength.
- Although NT incorporation led to increases in FS, the improvements were relatively limited compared to those observed in CS. This can be attributed to the fact that NT primarily enhanced matrix densification rather than crack-bridging mechanisms, which play a more dominant role in flexural behavior.
- In both CS and FS, the improvements achieved with 3% NT addition for both particle sizes were lower than those of the control mixture (P0). This reduction is attributed to particle agglomeration and increased water demand, which negatively affected dispersion and matrix homogeneity at high NT contents.
- SEM observations and ImageJ-assisted 2D threshold segmentation revealed that the control mixture (P0) exhibited larger and more interconnected surface void regions, indicating higher pore connectivity and limited matrix continuity.
- Incorporation of NT, particularly at 15–35 nm, significantly enhanced matrix densification and pore refinement, as evidenced by reduced surface void regions and more homogeneous C–S–H distribution. However, at 3% NT content, localized agglomeration increased microstructural heterogeneity and partially diminished the efficiency of pore refinement.
- The use of NT as a substitute enhanced sustainability by diminishing PC content while preserving or enhancing essential fresh, physical, and mechanical characteristics. The observed increases in CS, along with decreases in WA and AP, suggest that NT can be effectively employed to enhance material efficiency and durability, thereby advancing cementitious systems with reduced CO2 emissions.
- Future research should focus on evaluating the long-term durability of NT-modified mortars, including chloride penetration, sulfate resistance, and freeze–thaw behavior, and on performing life cycle assessments (LCA) to quantify environmental benefits. Moreover, examining NT dispersion tactics, hybrid nanomodification techniques, and the interaction of NT with additional cementitious materials would elucidate its role in sustainable cement-based materials.

## Data Availability

All data supporting the findings of this study are included within the article.

## Nomenclature

a_0_: Initial chromaticity coordinate in CIELAB system
a_t_: Chromaticity coordinate after UV exposure at time t
AP: Apparent porosity (%)
b_0_: Initial chromaticity coordinate in CIELAB system
b_t_: Chromaticity coordinate after UV exposure at time t
C_0_: Binder-material cost of control mixture (TL)
C_NT_: Binder-material cost of NT-modified mixture (TL)
CPI: Cost-performance index (−)
CS: Compressive strength (MPa)
CS_0_: Compressive strength of control mixture (MPa)
CS_NT_: Compressive strength of NT-modified mixture (MPa)
CS Gain: Compressive strength gain (%)
d: Curing age (days)
ΔE: Total color difference
FD: Flow diameter (mm)
FS: Flexural strength (MPa)
L_0_: Initial lightness value in CIELAB system
L_t_: Lightness value after UV exposure at time t
MB: Methylene blue
MPa: Megapascal
nm: Nanometer
NT: Nanotitanium dioxide
PC: Portland cement
RCI: Relative cost increase (%)
SEM: Scanning electron microscopy
SP: Superplasticizer
SSA: Specific surface area (m^2^ g^–1^)
t: UV irradiation time (h)
WA: Water absorption (%)
w/b: Water-to-binder ratio (−)

## References

[1] Bararjani H., Yahyaee T., Saradar A., Moein M. M., Tavakoli D. (2026). Characterisation
of Magnetic Properties in Cementitious Composites for Advanced Wireless
Power Transfer Systems Using Magnetic Sand, Magnetite Powder, GGBFS,
and Silica Fume. Results Eng..

[2] Yang J., Li T., Zeng J., Su Y., Lu S., Tian C., Strnadel B., He X. (2026). Development
of carbon sequestration
cement-based materials with mechanochemically carbonated carbide slag. J. Build. Eng..

[3] Yuan J., Chang J., Cui K. (2025). Development
of Highly Reactive Supplementary
Cementitious Materials via Carbonated Ternesite: Toward High Cement
Performance and CO2 Emission Reduction. ACS
Sustainable Chem. Eng..

[4] Zhang Y., Xu X., Ding S., Zhao Q., Chang J. (2026). Influence of Seawater-Derived
Salts on Ye’elimite Hydration Kinetics and Microstructural
Evolution: Toward Developing Sustainable Calcium Sulfoaluminate Cement-Based
Materials. ACS Sustainable Chem. Eng..

[5] Du M., Yang Y., Miao J., Hou R., Zhai K., Yao X. (2025). A deep learning-based study of the
role of graphene oxide nanosheets
on the microstructure of cement paste. ACS Appl.
Nano Mater..

[6] Li H., Zhao Y., Zhao Y., Zhang M., Niu Y., Cao X. (2025). Advances in the mechanism and application of nanoparticles in concrete
property modification. Inorganics.

[7] Chen Y., Zeng X., Zhang Y., Liu X., Xie Y., Guo H., Wang Y. (2025). Effects of nanomaterials
on early-age properties and
microstructure of calcium sulfoaluminate-Portland cement-based repair
mortar under-10°C curing. Case Stud. Constr.
Mater..

[8] Zeng H., Chen J., Luo X., Qu S., Li Y., Hu Y., Tian Y. (2025). Dispersibility of graphene-family
materials and their
impact on the properties of cement-based materials: Application challenges
and prospects. Rev. Adv. Mater. Sci..

[9] Vignesh J., Ramesh B., Xavier J. R. (2025). Recent advances
in nano-engineered
cement composites for sustainable and smart infrastructure. Nanotechnol. Environ. Eng..

[10] Feng S., Guan S. (2025). Influence of nano-SiO2 and nano-TiO2
on early hydration process of
cement: Hydration rate, hydration products microstructure, calcium
ion solubility, and diffusion ability. Constr.
Build. Mater..

[11] Guo E., Zhang W., Lai J., Hu H., Xue F., Su X. (2025). Enhancement of cement-based materials: Mechanisms, impacts, and applications
of carbon nanotubes in microstructural modification. Buildings.

[12] Chadha V., Singla S. (2025). A review on classification and effect
of nanoparticles
on workability, mechanical properties, durability, and microstructure
of cement composites. Iran. J. Sci. Technol.,
Trans. Civ. Eng..

[13] Javan H., Honarbakhsh A., Movahedifar S. M., Nobahari M., Zhiani R. (2025). The use of
dendritic fibrous nano-titanium to enhance the initial characteristics
and durability of lightweight concrete. Sci.
Rep..

[14] Bheel N., Chohan I. M., Alraeeini A. S., Alwetaishi M., Waheeb S. A., Alkhattabi L., Benjeddou O. (2025). Optimization
of durability characteristics of engineered cementitious composites
combined with titanium dioxide as a nanomaterial applying RSM modelling. Sci. Rep..

[15] Bica B. O., de Melo J. V. S. (2020). Concrete blocks
nano-modified with zinc oxide (ZnO)
for photocatalytic paving: Performance comparison with titanium dioxide
(TiO2). Constr. Build. Mater.

[16] Zhang H., Wang C. (2025). Research and improvement
of mechanical properties of cement nanocomposites
for well cementing. Nanotechnol. Rev..

[17] Kang M., Wang B., Liu C., Sun W., Li Q., Liu Z. (2025). Early hydration behavior and multi-scale performance
of nano-TiO2
modified fly ash cemented tailings backfill. Constr. Build. Mater..

[18] Moro C., Francioso V., Velay-Lizancos M. (2021). Impact of nano-TiO2 addition on the
reduction of net CO2 emissions of cement pastes after CO2 curing. Cem. Concr. Compos..

[19] Montes
Rubio T., Rosas Casarez C. A., Orozco Carmona V. M., Ahumada Cervantes R., Luna Valenzuela A., Cervantes Rosas M. D. L. A., Chinchillas Chinchillas M. D. J. (2025). Performance of Nanotechnology in
Cementitious Materials: Synthesis and Application. Materials.

[20] Raj P., Singh N., Singh P. (2025). Reviewing the degradation of environmental
pollution in cement concrete structures using Nano-Titanium Dioxide. J. Mater. Eng. Struct..

[21] Jamjala S., Thulasirangan Lakshmidevi M., Karthik Reddy K. S. K., Kafle B., Al-Ameri R. (2025). A critical review on synergistic
integration of nanomaterials in 3D-Printed concrete: rheology to microstructure
and eco-functionality. Appl. Sci..

[22] American Society for Testing and Materials. Standard practice for mechanical mixing of hydraulic cement pastes and mortars of plastic consistency ASTM International: 2022.

[23] American Society for Testing and Materials. Standard test method for flow of hydraulic cement mortar ASTM International: 2022.

[24] American Society for Testing and Materials. Standard test method for density, absorption, and voids in hardened concrete ASTM International: 2022.

[25] ASTM International. ASTM C1378 – Standard test method for determination of resistance to staining. ASTM International, 2022.

[26] American Society for Testing and Materials. Standard test method for flexural strength of hydraulic-cement mortars. ASTM International: 2022.

[27] American Society for Testing and Materials. Standard test method for compressive strength of hydraulic-cement mortars (using portions of prisms broken in flexure) ASTM International: 2022.

[28] Nisar N., Rahman I., Paruthi S., Khan A. H., Sabi E., Alyaseen A. (2025). Enhancing concrete properties with nano-SiO2 and nano-TiO2:
a review. J. Struct. Integr. Maint..

[29] Essawy A. A., Aleem S. A. E. (2014). Physico-mechanical
properties, potent adsorptive and
photocatalytic efficacies of sulfate resisting cement blends containing
micro silica and nano-TiO2. Constr. Build. Mater..

[30] Senff L., Hotza D., Lucas S., Ferreira V. M., Labrincha J. A. (2012). Effect
of nano-SiO2 and nano-TiO2 addition on the rheological behavior and
the hardened properties of cement mortars. Mater.
Sci. Eng. A.

[31] Mohseni E., Miyandehi B. M., Yang J., Yazdi M. A. (2015). Single and combined
effects of nano-SiO2, nano-Al2O3 and nano-TiO2 on the mechanical,
rheological and durability properties of self-compacting mortar containing
fly ash. Constr. Build. Mater..

[32] Liao G., Yao W., She A., Shi C., Zuo J., Wu D. (2023). Interfacial
design of nano-TiO2 modified recycled concrete powder for building
self-cleaning. Colloids Surf., A.

[33] Döndüren M. S., Al-Hagri M. G. (2022). A review
of the effect and optimization of use of nano-TiO2
in cementitious composites. Res. Eng. Struct.
Mater..

[34] Yousefi A., Allahverdi A., Hejazi P. (2013). Effective dispersion of nano-TiO2
powder for enhancement of photocatalytic properties in cement mixes. Constr. Build. Mater..

[35] Joshaghani A., Balapour M., Mashhadian M., Ozbakkaloglu T. (2020). Effects of
nano-TiO2, nano-Al2O3, and nano-Fe2O3 on rheology, mechanical and
durability properties of self-consolidating concrete (SCC): An experimental
study. Constr. Build. Mater..

[36] Wang Z., Shen Y., Li Y., Du H. (2025). Workability and Mechanical
Performances of Cement Paste with Nano-TiO2. J. Wuhan Univ. Technol., Mater. Sci. Ed..

[37] Zhong C., Yu Z., Zhou J., Long Y., Tian P., Chen J. (2022). Effect of
nano-TiO2 on capillary water absorption of recycled aggregate concrete. Coatings.

[38] Shafaei D., Yang S., Berlouis L., Minto J. (2020). Multiscale
pore structure
analysis of nano titanium dioxide cement mortar composite. Mater. Today Commun..

[39] Dantas S. R. A., Serafini R., de Oliveira Romano R.
C., Vittorino F., Loh K. (2019). Influence of the nano TiO2 dispersion procedure on fresh and hardened
rendering mortar properties. Constr. Build.
Mater..

[40] Ma B., Li H., Li X., Mei J., Lv Y. (2016). Influence of nano-TiO2
on physical and hydration characteristics of fly ash–cement
systems. Constr. Build. Mater..

[41] Akono A. T. (2020). Effect
of nano-TiO2 on C–S–H phase distribution within Portland
cement paste. J. Mater. Sci..

[42] Kadhim M. J., Al-Jadiri R. S., Wahab Ali M. A. A. (2019). Study the effect of addition nano-TiO2
by dispersion method on the some mechanical properties and durability
of cement mortar. IOP Conf. Ser. Mater. Sci.
Eng..

[43] Pozo-Antonio J. S., Dionísio A. (2017). Physical-mechanical
properties of mortars with addition
of TiO2 nanoparticles. Constr. Build. Mater.

[44] Xu Z., Gao J., Zhou Z., Zhao Y., Chen X. (2020). Hydration and microstructure
of tricalcium silicate incorporating nano-TiO2. Constr. Build. Mater..

[45] Ying J., Zhou B., Xiao J. (2017). Pore structure and chloride diffusivity
of recycled aggregate concrete with nano-SiO2 and nano-TiO2. Constr. Build. Mater..

[46] Shaaban I., El-Sayad H. I., El-Ghaly A. E., Moussa S. (2020). Effect of micro TiO_2_ on cement mortar. Eur. J. Mater. Sci.
Eng..

[47] Ziada M. (2025). The effect
of nano-TiO2 and nano-Al2O3 on mechanical, microstructure properties
and high-temperature resistance of geopolymer mortars. Arabian J. Sci. Eng..

[48] Rao S., Silva P., De Brito J. (2015). Experimental
study of the mechanical
properties and durability of self-compacting mortars with nano materials
(SiO2 and TiO2). Constr. Build. Mater..

[49] Noorvand H., Ali A. A. A., Demirboga R., Farzadnia N., Noorvand H. (2013). Incorporation of nano TiO2 in black
rice husk ash mortars. Constr. Build. Mater..

[50] Duan P., Yan C., Luo W., Zhou W. (2016). Effects of adding nano-TiO2 on compressive
strength, drying shrinkage, carbonation and microstructure of fluidized
bed fly ash based geopolymer paste. Constr.
Build. Mater..

[51] Lee B. Y., Jayapalan A. R., Kurtis K. E. (2013). Effects of nano-TiO2 on properties
of cement-based materials. Mag. Concr. Res..

[52] Ren J., Lai Y., Gao J. (2018). Exploring the influence of SiO2 and TiO2 nanoparticles
on the mechanical properties of concrete. Constr.
Build. Mater..

[53] Chunping G., Qiannan W., Jintao L., Wei S. (2018). The effect of nano
TiO2 on the durability of ultra-high-performance concrete with and
without a flexural load. Ceram.-Silik..

[54] Ng D. S., Paul S. C., Anggraini V., Kong S. Y., Qureshi T. S., Rodriguez C. R., Liu Q.-f., Šavija B. (2020). Influence
of SiO2, TiO2 and Fe2O3 nanoparticles on the properties of fly ash
blended cement mortars. Constr. Build. Mater..

[55] Zhang Y., Wang G., Huang B., Liu F., Qu F., Zhu M. (2025). Mechanical characteristics, thermal
reflective performance and energy-saving
efficiency of nano TiO2 cement mortars for building envelops. J. Build. Eng..

[56] Othman F. M., Abdul Hameed A. A., Ibrahim S. I. (2016). Studying the effect of nano additives
and coating on some properties of cement mortar mixes. Eng. Technol. J..

[57] Khalil M. M., Hisham M., El-Azab I. A. (2025). Enhanced
flexural behavior of reinforced
concrete beam containing nano-titanium and glass powder, along with
novel ductile bars. Comput. Concr..

[58] Hua Y., Wang G., Liu F., Huang B., Zhu M., Qu F. (2025). Investigation on strength,
drying shrinkage and photocatalytic efficiency
of TiO2 cement composite mortar by the layering method. KSCE J. Civ. Eng..

[59] Kalinowski M., Chilmon K., Jackiewicz-Rek W. (2025). Photocatalytic
performance of cementitious
composites modified with second-generation nano-tio2 dispersions:
influence of composition and granulation on nox purification efficiency. Coatings.

[60] Liu H., Wang Y., Wang X., Liu R., Zhang P. (2025). TiO2 Nanoparticles
in Soil: Adsorption, Transformation, and Environmental Risks. Powders.

[61] Sarina S., Waclawik E. R., Zhu H. (2013). Photocatalysis on supported gold
and silver nanoparticles under ultraviolet and visible light irradiation. Green Chem..

[62] Simanjuntak M., Joni I. M., Faizal F., Gultom N. S., Kuo D.-H., Panatarani C. (2025). Photoluminescence
of oxygen vacancy-rich nano-TiO2
photocatalyst for methylene blue color degradation. Mater. Sci. Eng., B.

[63] Coffetti D., Crotti E., Coppola L. (2023). Long-term
properties of self-cleaning
alkali-activated slag-based mortars with titanium dioxide nanoparticles. Constr. Build. Mater..

[64] Tyukavkina V. V., Shchelokova E. A., Tsyryatyeva A. V., Kasikov A. G. (2021). TiO2–SiO2
nanocomposites from technological wastes for self-cleaning cement
composition. J. Build. Eng..

[65] Lindgreen H., Geiker M., Krøyer H., Springer N., Skibsted J. (2008). Microstructure
engineering of Portland cement pastes and mortars through addition
of ultrafine layer silicates. Cem. Concr. Compos..

[66] Seifi S., Levacher D., Razakamanantsoa A., Sebaibi N. (2023). Microstructure of dry
mortars without cement: specific surface area, pore size and volume
distribution analysis. Appl. Sci..

[67] Aodkeng, S. ; Sinthupinyo, S. ; Hanpongpun, W. ; Chaipanich, A. Microstructure, Interface Characteristics and Compressive Strength of CNTs/Clay Hybrid-High Early Strength Portland Cement Composites. Ceramics International: 2025.

[68] Avinash
Reddy N., Sri Chandana P. (2025). Critical analysis of the compact
microstructural properties of ordinary Portland and Portland Pozzolana
cement mortars incorporated with nano-TiO2. J. Build. Pathol. Rehabil..

[69] Feng S., Xiao H., Guan S. (2025). Influence
of Nano-SiO2 and Nano-TiO2
on properties and microstructure of cement-based materials. Constr. Build. Mater..

[70] Srivastava A., Mishra A., Singh S. K. (2025). Effect of nano TiO2 and reinforcement
of polypropylene fiber in standard concrete: an experimental and numerical
approach. Can. J. Civ. Eng..

[71] Goyal R., Verma V. K., Singh N. B. (2025). Hydration
and stain resistance of
blended cements in presence of nano-TiO2. Innov.
Infrastruct. Solut..

[72] Theja A. R., Sivaramakrishnaiah M., Gopala Krishna Sastry K. V. S., Sashidhar C., Paramasivam P., Kanti P. K., Ayani A. G. (2025). Influence of Nano
Titanium Dioxide on Strength and Durability of Ambient-Cured GGBS-Based
Geopolymer Concrete. Results Eng..

[73] Rahman I., Paruthi S., Dev N., Arif M., Khan A. H., Hasan M. A., Prasad C. V. S. R., Alyaseen A. (2025). Mechanical, microstructure,
durability, and economic assessment of nano titanium dioxide integrated
concrete. Sci. Rep..

[74] Sekar S. K., Ranjith R., Prasanth S. (2025). Sustainable concrete
matrix using
ceramic electrical insulator waste as binder and filler-performance
under elevated temperature. World J. Eng..

[75] Montazeri-Pour M., Fallahpisheh O., Rajabi M. (2025). Preparation and characterization
of core-shell structured CaCO3-SiO2 and hollow silica nanoparticles
and evaluation of their effects on cement mortar properties. J. Sustainable Cem.-Based Mater..

[76] Leelatanon S., Setkit M., Imjai T., Ghorbel E., Kim B. (2025). High performance
synthetic fiber-reinforced concrete mixed with nanoparticles: A proof-of-concept
green railway sleeper product. Sustainable Constr..

[77] Vaid U., Balwinder Lallotra D. (2024). Effect on
concrete strength and durability with partial
replacement of cement by Nano-titanium dioxide (nano-TiO2) and ground
granulated blast furnace slag (GGBS): A Review Summary. IOP Conf. Ser.: Earth Environ. Sci..

[78] Ashwini R. M., Sambhana K. D., Manjunatha M. (2025). Enhancing
concrete performance and
sustainability with nano tio_2_: engineering, microstructural,
and life cycle evaluation. Iran. J. Sci. Technol.,
Trans. Civ. Eng..

[79] Nagaraj K., Radha S., Deepa C. G., Raja K., Umapathy V., Badgujar N. P., Parekh N. M., Manimegalai T., Archana Devi L., Uthra C. (2025). Photocatalytic advancements
and applications
of titanium dioxide (TiO_2_): Progress in biomedical, environmental,
and energy sustainability. Next Res..

[80] Prashanth, S. ; Singh, S. K. ; Pongen, R. ; Verma, Y. K. ; Vijay, K. Investigation of Mechanical Properties and Terminological Performance of TiO2 Filled Hybrid GFRP Composite and Nanocomposite—A Review Technological Advancements in Engineering and Management Sciences Springer 2026 83–102 10.1007/978-3-032-04606-2_7

